# An Eggplant Recombinant Inbred Population Allows the Discovery of Metabolic QTLs Controlling Fruit Nutritional Quality

**DOI:** 10.3389/fpls.2021.638195

**Published:** 2021-05-17

**Authors:** Maria Sulli, Lorenzo Barchi, Laura Toppino, Gianfranco Diretto, Tea Sala, Sergio Lanteri, Giuseppe Leonardo Rotino, Giovanni Giuliano

**Affiliations:** ^1^Italian National Agency for New Technologies, Energy and Sustainable Economic Development (ENEA), Casaccia Research Centre, Rome, Italy; ^2^Department of Agricultural, Forest and Food Sciences (DISAFA), Plant Genetics and Breeding, University of Turin, Grugliasco, Italy; ^3^CREA, Council for Agricultural and Economics Research, Research Centre for Genomics and Bioinformatics, Montanaso Lombardo, Italy

**Keywords:** *Solanum melongena* (L.), metabolic profiling, glycoalkaloids, anthocyanins, polyamine conjugates

## Abstract

Eggplant (*Solanum melongena* L.) represents the third most important crop of the Solanaceae family and is an important component of our daily diet. A population of 164 F6 recombinant inbred lines (RILs), derived from two eggplant lines differing with respect to several key agronomic traits, “305E40” and “67/3,” was grown to the commercial maturation stage, and fruits were harvested, separated into peel and flesh, and subjected to liquid chromatography Liquid Chromatography/Mass Spectrometry (LC/MS) analysis. Through a combination of untargeted and targeted metabolomics approaches, a number of metabolites belonging to the glycoalkaloid, anthocyanin, and polyamine classes and showing a differential accumulation in the two parental lines and F1 hybrid were identified. Through metabolic profiling of the RILs, we identified several metabolomic quantitative trait loci (mQTLs) associated with the accumulation of those metabolites. Each of the metabolic traits proved to be controlled by one or more quantitative trait loci (QTLs); for most of the traits, one major mQTL (phenotypic variation explained [PVE] ≥ 10%) was identified. Data on mQTL mapping and dominance–recessivity relationships of measured compounds in the parental lines and F1 hybrid, as well as an analysis of the candidate genes underlying the QTLs and of their sequence differences in the two parental lines, suggested a series of candidate genes underlying the traits under study.

## Introduction

Eggplant (*Solanum melongena L*.) is a diploid species (2*n* = 2*x* = 24) belonging to the Solanaceae family, genus *Solanum* and subgenus *Leptostemonum*. It has originated in Africa and was probably domesticated in Asia (Weese and Bohs, 2010), where it has been cultivated for over 1,500 years. Cultivated eggplant represents the third most important crop of the Solanaceae family after potato and tomato; it is cultivated worldwide, with a global production of 54 Mt in 2018 (FAOSTAT^1^) China, India, Iran, and Indonesia are the leading producing countries, while Egypt, Turkey, and Italy are the main producers in the Mediterranean region. Two other *Solanum* species, native from Africa, are commonly cultivated: the scarlet eggplant (*Solanum aethiopicum* L.) and the gboma eggplant (*Solanum macrocarpon* L.), with which eggplant is fully cross-compatible.

Eggplant fruits differ strongly in size, shape, and skin color. Most of their nutritional properties are related to their content in phenolics, especially chlorogenic and hydroxycinnamic acids and their conjugates, as well as in the skin of pigmented genotypes, anthocyanins, and other phenylpropanoids (Whitaker and Stommel, 2003; Mennella et al., 2012).

Eggplant genetic maps have been constructed from both interspecific (Doganlar et al., 2002a,b; Frary et al., 2003; Wu et al., 2009) and intraspecific crosses (Barchi et al., 2010, 2012; Fukuoka et al., 2012). These have been used to map quantitative trait loci (QTLs) controlling morphological and domestication traits, including fruit weight, shape, and color (Portis et al., 2014), disease resistances (Barchi et al., 2018), and some biochemical features (Toppino et al., 2016). Finally, an association panel of 191 accessions, genotyped with the restriction site associated DNA (RAD) markers developed by Barchi et al. (2011) and mapped by Barchi et al. (2012), was recently employed for a genome-wide association (GWA) approach for identifying genomic regions involved in anthocyanin and many other traits of agronomic interest (Cericola et al., 2014; Portis et al., 2015). To this date, however, few systematic efforts have been conducted to map QTLs controlling fruit metabolic composition (Gramazio et al., 2014; Toppino et al., 2016).

A chromosome-anchored eggplant genome sequence has been recently published (Barchi et al., 2019). This sequence, in combination with the technical advances in metabolic profiling (Fiehn et al., 2000; Schauer et al., 2006), creates new opportunities to study the genetic basis of eggplant fruit nutritional quality.

**Figure 1:**
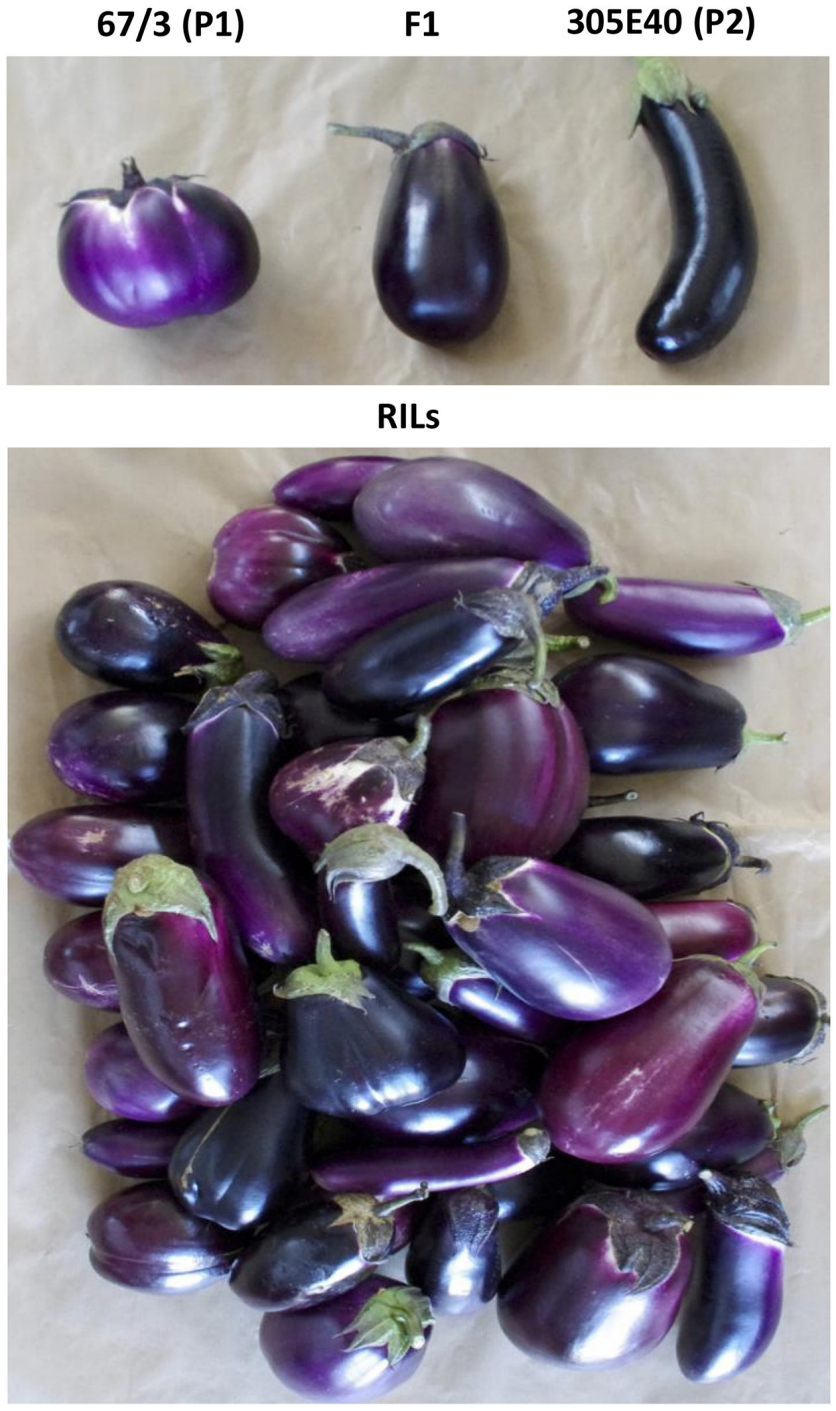
Phenotypes of fruits at commercial maturation. Top: the two parents “67/3” (P1, male) and “305E40” (P2, female) of the RIL population, and the F1 hybrid. Bottom: the F6 RILs.

We undertook the metabolic characterization of fruits of an eggplant recombinant inbred line (RIL) population (Toppino et al., 2020). The population consists of 164 F6 RILs, obtained by crossing the breeding line “305E40,” a doubled haploid derivative of an interspecific somatic hybrid between *S. aethiopicum* and *S. melongena*, and the breeding line “67/3,” an F6 selection from an intraspecific *S. melongena* cross. The female parent “305E40” produces long, highly pigmented dark purple fruit, while the male parent “67/3” produces round lilac fruits (Figure 1). The “67/3” genotype has been used for the generation of a high-quality genome sequence, and the “305E40” line has been re-sequenced (Barchi et al., 2019). First, metabolites that differentially accumulated in the two parental lines and the F1 hybrid were identified through an untargeted approach and a combination of accurate mass, isotopic patterns, MS^2^ profiles, and comparison with authentic standards. Then, additional metabolites belonging to the same metabolic pathways were identified through targeted approaches. Both untargeted and targeted metabolites were quantified in the RILs, leading to the discovery of metabolic QTLs controlling their levels.

## Materials and Methods

### Plant Material

An F6 RIL population, bred from a cross between lines “305E40” and “67/3,” was field grown, along with both parents and the F1 hybrid, in the field at Montanaso Lombardo (45°20′N, 9°26′E). Each individual RIL progeny was sown in the greenhouse, and plantlets were transplanted in an open field at the four- to five-leaf stage. One leaflet per RIL was stored at −80°C for DNA extraction. The material was arranged as a set of two randomized complete blocks with four plants per entry per block. For fruit sampling, at least four different fruits were collected from four different plants for each biological replicate at the commercial ripening stage B (~30 days after flowering [DAF]) as described by Mennella et al. (2012). A 2-mm-deep layer comprising the peel was carefully harvested using a sharp knife peel, taking slices of about 4 cm^2^ from opposite sides of the fruits to pool pieces of peel tissues that were exposed to different sunlight intensities. Pulp was reduced to square slices 1 cm thick, removing the seeds. Tissues were frozen in liquid nitrogen and stored at −80°C.

### Polar Extraction and LC/ESI/MS Analysis

Frozen tissues were freeze-dried and ground in 2 ml Eppendorf tubes with a tungsten bead using a TissueLyser (Qiagen) at 30 Hz for 30 s. Five milligrams of powder from either flesh or peel were extracted with 1.5 ml of 75% methanol/0.05% v/v trifluoroacetic acid, spiked with 3 μg/L formononetin (Sigma-Aldrich) as an internal standard. After vortexing for 30 s and centrifugation (15 min at 20,000 g, 15°C), 0.6 ml of the supernatant was removed and transferred into filter (PTFE) vials for LC/MS analysis (Waters). Ten microliters of filtered extract were injected. LC analysis was performed using a C18 Luna column (Phenomenex, Macclesfield, UK), 2.0 × 150 mm, 2.5 μm particle size. Total run time was 32 min using an elution system running at 0.250 ml/min and consisting of (A) water (0.1% formic acid and 10 pg/ml caffeine) and (B) acetonitrile:H_2_O 90:10 (0.1% formic acid and 10 pg/ml caffeine as an internal mass reference, to improve mass accuracy). Gradient was 0 to 0.5 min 95% A/5% B, 24 min 25% A/75% B, and 26 min 95% A/5%. The MS analysis was performed using an LTQ Orbitrap Discovery mass spectrometer using an ESI, with an FTMS *m*/*z* range of 110–1,800 and source operating in positive ion mode (resolution 30,000), which was the one found, in preliminary trials, to give the highest number of differentially abundant (DA) metabolites.

Parameters were capillary temperature 270°C; sheath and auxiliary gas set at, respectively, 50 and 5 units; spray voltage 4.5 kV, capillary voltage set at 10 V, and tube lens at 80 V. All the chemicals and solvents used during the entire procedure were of LC/MS grade (Chromasolv).

### Untargeted and Targeted Analysis of Polar Metabolites

Untargeted analysis of DA metabolites was carried out using the SIEVE software (v1.2, Thermo Fisher Scientific), which performs chromatogram alignment, peak picking, and public database (e.g., ChemSpider, KEGG, PubChem, and PlantCyc databases) querying based on accurate masses (*m*/*z*). LC/MS data were processed, applying a “control compare trend” type of experiment for “small molecules,” using an already-described method (Coppola et al., 2019). With this automated analysis, MS intensities from raw LC/MS data were processed, grouping MS chromatograms as replicates assigned to each sample and generating a list of “frames” for each group of peaks found in the samples, within a specified *m*/*z* value and retention time. Frames showing intensity value differences of more than 2-fold, with a *p* ≤ 0.05, between the two parental lines or between the F1 hybrid and either of the parental line were selected. Compounds were tentatively identified based on accurate mass (*m*/*z*) in full-scan MS (M+H+ or M+) and isotopic ratios (level C); MS^2^ spectra generated by data-dependent MS/MS, compared to public (KEGG, Metlin, and PubChem), in-house, or *in silico* generated MS^2^ spectra (Ruttkies et al., 2016) (level B); and co-migration with authentic standards (level A), using the Xcalibur software 4.4.16 Qual browser (Thermo Fisher Scientific, USA). Authentic standards were purchased from Sigma-Aldrich. The identification levels of each metabolite are described in Supplementary Table 1, and examples of MS^2^ identification are given in the Supplementary Text and Figures. For pathway walking, metabolites metabolically related to the ones initially identified through untargeted analysis were identified based on accurate mass (*m*/*z*), isotopic ratios, and, when available, MS^2^ data (Supplementary Table 1). Relative levels of accumulation of investigated metabolites were calculated as fold average and the standard deviation of integrated areas under the *m*/*z* peak of the adduct of each metabolite and the internal standard peak area (Fold/ISTD), using the TraceFinder 4.1 software (Thermo Fisher Scientific, USA), using two biological replicates (Coppola et al., 2019).

### Statistical Analyses and mQTL Detection

Statistical analyses were performed using the R software (R Team, 2013). A conventional analysis of variance was applied to estimate genotype and environment effects based on the linear model *Y*_*ij*_ = μ + *g*_*i*_ + *b*_*j*_ + *e*_*ij*_, where μ, *g, b*, and *e* represent, respectively, the overall mean, the genotypic effect, the replicate effect, and the error. Broad-sense heritability values were given by σ^2^*G*/([σ^2^*G* + σ^2^*E*]/*n*), where σ^2^*G* represented the genetic variance, σ^2^*E* the residual variance, and *n* the number of replicates. Correlations between traits were estimated using the Spearman coefficient, and kurtosis and skewness were calculated. Segregation was considered as transgressive when at least one individual RIL recorded a trait value higher or lower by at least two standard deviations than the higher or lower scoring parental line. A recently developed single-nucleotide polymorphism (SNP)-based map (Toppino et al., 2020) was used for QTL detection. Briefly, library construction was carried out using the *Hin*dIII–*Mse*I enzyme combination followed by a biotin/streptavidin-coated beads-based purification step. DNA libraries were pooled and sequenced on an Illumina HiSeq 2500 platform (Illumina Inc., San Diego, CA, USA), using 150 PE chemistry and following the manufacturer protocol at Biodiversa srl (Rovereto, TN, Italy). Overall, the map includes 7,249 SNP markers and spans 2,169 cM.

A multiple QTL mapping (MQM) method (Jansen, 1993, 1994), as implemented in the MapQTL v4 software (Van Ooijen, 2004) was used for QTL analysis. QTLs were initially identified using interval mapping; afterwards, one linked marker per putative QTL was treated as a cofactor in the approximate multiple QTL model. Cofactor selection and MQM analysis were repeated until no new QTL could be identified. LOD thresholds for declaring a QTL to be significant at the 5% genome-wide probability level were established empirically by applying 1,000 permutations per trait (Churchill and Doerge, 1994). Additive genetic effect and the percentage of the phenotypic variation explained (PVE) by each QTL were obtained from the final multiple QTL model. Individual QTLs were prefixed by a trait abbreviation, followed by the relevant chromosome designation. The confidence interval of the QTL was calculated as the LODmax^−1^ interval or at least by considering 0.3 Mb upstream and downstream (if not differently reported in the text) of the marker identified at the QTL.

Based on the annotation v3.0, the confidence intervals of QTLs were analyzed with the SnpEff v4.3 program (Cingolani et al., 2012) to infer any potential deleterious effect on candidate genes for the metabolites in study. Briefly, resequencing data of the parental line “305E40” (Barchi et al., 2019) were aligned against the reference “67/3” eggplant genome, using the Burrows-Wheeler Aligner (BWA) (Li, 2013) (i.e., mem command) with default parameters and avoiding multiple-mapping reads. BAM files were processed and used for SNP calling using bcftools mpileup/call/norm utilities (Li, 2011) with default parameters, except for the use of the multiallelic calling model (–m option), minimum mapping quality (*Q* = 20), and the filtering out of multimapping events (–*q* > 1).

The effect of each SNP/indel was classified into four classes of effects: (1) modifier effect, as variants located outside genes (non-transcribed regions or introns); (2) low effect, as synonymous variants in coding regions; (3) moderate effect, as variants altering the aminoacidic sequence; and (4) high effect, as variants changing frameshift, thereby introducing/eliminating stop codons or modifying splice sites. For variant annotation, the CMplot^2^ was used for drawing QTL results.

## Results

### Untargeted Metabolic Profiling of Fruits From the Parental Lines and the F1 Hybrid

**Figure 2:**
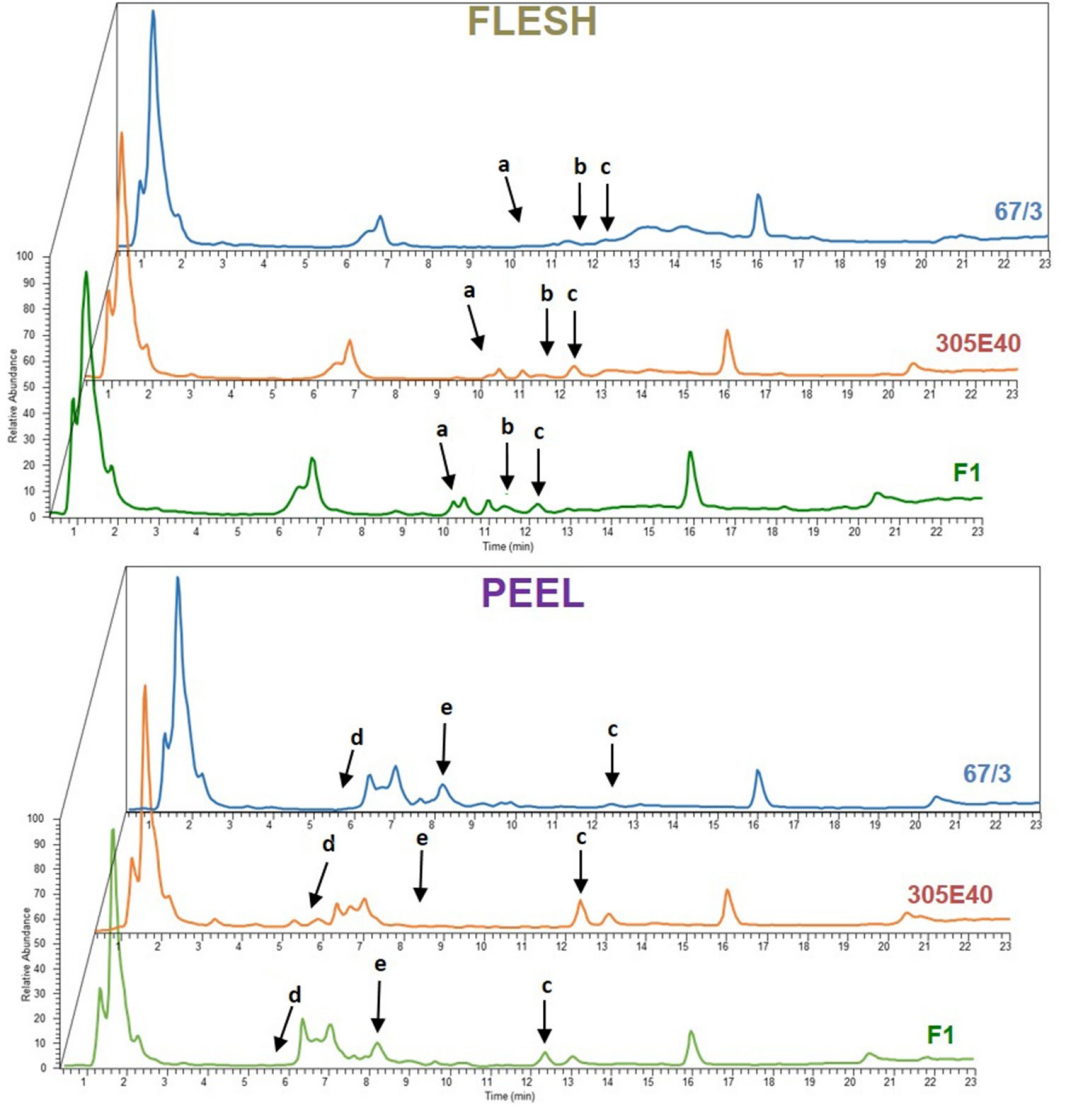
Representative Total Ion Current (TIC) chromatograms of flesh and peel samples from parental lines “67/3” (blue), “305E40” (orange), and F1 hybrid (green). Arrows indicate differentially expressed peaks: a, solamargine; b, malonylsolamargine; c, pseudoprotodioscin; d, delphinidin-3-rutinoside; e, nasunin.

The RIL population composed of 164 F6 RILs (Toppino et al., 2020), as well as the two parental lines “305E40” (P1, female parent) and “67/3” (male parent) and the F1 hybrid (HF1), were grown in an experimental field at Montanaso Lombardo (45°20′N, 9°26′E) in two independent, randomized blocks, each constituting a biological replicate. Fruits were harvested from each of the two blocks at the commercial ripe (B) stage (~30 DAF) (Mennella et al., 2012), separated into peel and flesh fractions, and freeze-dried. Polar metabolites from flesh and peel were extracted from parental lines and the HF1 hybrid as previously described and analyzed by LC/ESI/MS analysis (see Materials and Methods). MS spectra collected were analyzed by performing an untargeted search for DA metabolites between “305E40” and “67/3” parents and the F1 hybrid. To this end, the MS data from the three genotypes were analyzed in all possible pairwise comparisons using the SIEVE software v1.2 (Thermo Fisher Scientific). An example of the chromatograms is shown in Figure 2. Peaks showing more than a 2-fold change between the two parental lines or between the F1 hybrid and either of the parental line with a *p* ≤ 0.05 were classified as DA and are shown in the “untargeted” section of Table 1.

**Table 1:** Differentially abundant metabolites in flesh and peel extracts of the parental lines, the F1 hybrid and the RIL populations.

	**Metabolite**	**Code**	**67/3 (P1)**	**305E40 (P2)**	**F1**	**cv**	**FC P2/P1**	**FC F1/P1**	**FC HF1/P2**	**Inh**	**RILs**
***FLESH***											
Untargeted	Pseudoprotodioscin	*F-PPROT*	0.05 ± 0.01	0.18 ± 0.04	0.07 ± 0.01	0.57	3.6^**^	1.4^**^	0.38^**^	R	0.12 ± 0.06
	Solamargine	*F-SOLM*	0.04 ± 0.005	0.09 ± 0.02	0.07 ± 0.02	1.83	2.25^**^	1.75^**^	0.78	D	0.18 ± 0.33
	Malonyl-solamargine	*F-MSOLM*	0.05 ± 0.02	0.08 ± 0.03	0.13 ± 0.03	1.55	1.6	2.6^**^	1.62^*^	O	0.13 ± 0.21
	n,n'-dicaffeoylspermidine	*F-DCASP*	ND	0.24 ± 0.02	0.06 ± 0.01	0.98	^**^	^**^	0.25^**^	S	0.13 ± 0.13
	n,n'-dicaffeoylspermidine iso	*F-DCASP2*	ND	0.11 ± 0.01	0.03 ± 0.01	0.96	^**^	^**^	0.27^**^	S	0.07 ± 0.06
Targeted	Demissine	*F-DEM*	0.01 ± 0.001	ND	0.003 ± 0.001	1.46	^**^	0.3^*^	^**^	D	0.00
	Solasonine	*F-SOLA*	0.03 ± 0.01	0.04 ± 0.01	0.08 ± 0.03	1.80	1.3	2.6^**^	2^**^	O	0.09 ± 0.16
***PEEL***											
Untargeted	Delphinidin-3-rutinoside	*P-D3R*	0.02 ± 0.002	0.29 ± 0.09	0.02 ± 0.001	1.16	14.5^**^	1	0.07^**^	R	0.25 ± 0.29
	Nasunin	*P-NAN*	0.57 ± 0.10	ND	0.56 ± 0.07	0.78	^**^	0.98	^**^	D	0.36 ± 0.28
	Rutin	*P-RUT*	0.01 ± 0.001	0.30 ± 0.04	0.02 ± 0.002	0.56	30^**^	2^*^	0.07^**^	R	0.02 ± 0.01
	Pseudoprotodioscin	*P-PPROT*	0.05 ± 0.01	0.43 ± 0.09	0.15 ± 0.04	0.55	8.6^**^	3^**^	0.35^**^	S	0.18 ± 0.10
	n,n'-dicaffeoylspermidine	*P-DCASP*	0.01 ± 0.002	0.13 ± 0.04	0.04 ± 0.01	0.90	13^**^	4^**^	0.31^**^	S	0.07 ± 0.06
	n-caffeoylputrescine	*P-CAPTR*	0.17 ± 0.05	0.65 ± 0.11	0.17 ± 0.04	0.50	3.82^**^	1	0.26^**^	R	0.17 ± 0.09
	n-dihydrocaffeoyl-n'-caffeoylspermidine	*P-DHCNCS*	0.13 ± 0.03	0.64 ± 0.18	0.31 ± 0.08	0.72	4.92^**^	2.38^**^	0.48^**^	S	0.69 ± 0.50
	n-coumaroylputrescine	*P-COPTR*	0.01 ± 0.002	0.12 ± 0.03	0.01 ± 0.003	0.91	12^**^	1^*^	0.08^**^	R	0.06 ± 0.06
	Glutamine	*P-GLUT*	0.08 ± 0.03	0.17 ± 0.05	0.05 ± 0.01	0.97	2.1^*^	0.6	0.29^**^	U	0.08 ± 0.08
	Unknown	*P-UNKN*	0.07 ± 0.01	0.15 ± 0.04	0.08 ± 0.02	0.66	2.14^**^	1.14	0.53^**^	R	0.56 ± 0.37
	Trigonelline	*P-TRIG*	0.17 ± 0.03	0.08 ± 0.02	0.06 ± 0.01	0.54	0.4^**^	0.35^**^	0.75	R	0.05 ± 0.03
	Acetylcholine	*P-ACH*	0.48 ± 0.05	0.08 ± 0.02	0.22 ± 0.02	0.58	0.16^**^	0.46^**^	2.75^**^	S	0.26 ± 0.15
Targeted	delphinidin 3-O-D-glucoside-5-(6-coumaroyl-D-glucoside)	*P-D3G5CG*	0.04 ± 0.01	ND	0.03 ± 0.004	0.59	^**^	0.75^**^	^**^	S	0.05 ± 0.03
	Kaempferol 3-O-beta-D-sophoroside	*P-KSOPH*	ND	0.03 ± 0.003	0.01 ± 0.002	0.73	^**^	^**^	0.33^**^	R	0.03 ± 0.02
	kaempferol-3-O-D-glucoside	*P-K3G*	0.003 ± 0.001	0.02 ± 0.01	0.006 ± 0.002	0.90	6.7^**^	2^*^	0.30^**^	S	0.03 ± 0.03

*Metabolite codes, averages, standard deviations (SD), coefficients of variation (cv), fold changes (FC;*

* *significant at p-value <0.05*,

** *significant at p-value < 0.01), and the inheritance (Inh.) in the F1 hybrid (D, Dominant; S, Semidominant; R, Recessive; O, overdominant; U, under-recessive) are shown. ND, not detectable*.

In the flesh, two main classes of DA metabolites were identified: (a) steroidal glycoalkaloids (SGAs)/saponins (SGAs: pseudoprotodioscin, solamargine, and malonyl solamargine) and (b) polyamine conjugates. Most metabolites showed higher relative levels in the “305E40” parent, especially pseudoprotodioscin, while both isomers of *n, n*′-dicaffeoylspermidine were not detectable in the “67/3” parent.

A higher number of DA metabolites were identified in peels: (a) glycosylated flavonols and anthocyanins (rutin, delphinidin-3-rutinoside, and nasunin); (b) one SGA (pseudoprotodioscin); (c) polyamine conjugates (*n, n*′-dicaffeoylspermidine, *n*-caffeoylputrescine, *n*-dihydrocaffeoyl-*n*′-caffeoylspermidine, and *p*-coumaroylputrescine); (d) amino acid glutamine; and (e) acetylcholine and trigonelline. Again, all compounds, except nasunin, trigonelline, and acetylcholine, showed higher relative levels in the “305E40” parent.

Depending on the compound, identification was based on accurate mass, isotopic patterns, MS^2^ fragmentation patterns based on data from literature or *in silico* prediction, or comparison with authentic standards (Supplementary Table 1 and Supplementary Text and Figures). Metabolites were defined as overdominant, dominant, semidominant, recessive, or under-recessive, according to the metabolite levels found in the F1 hybrid compared to those found in the two parental lines (Table 1).

### Pathway Walking

In order to better understand the possible nature of the mutations underlying the differences between the two parental lines, we decided to identify and measure, in the MS data, metabolites that are located on the same biosynthetic pathways with respect to the ones initially identified by the untargeted analysis. We call this approach “pathway walking.” Through this approach, we identified two additional DA SGAs in the flesh and three DA flavonoids (one anthocyanin and two glycosylated flavonols) in the peel (shown in the “targeted” section of Table 1).

**Figure 3:**
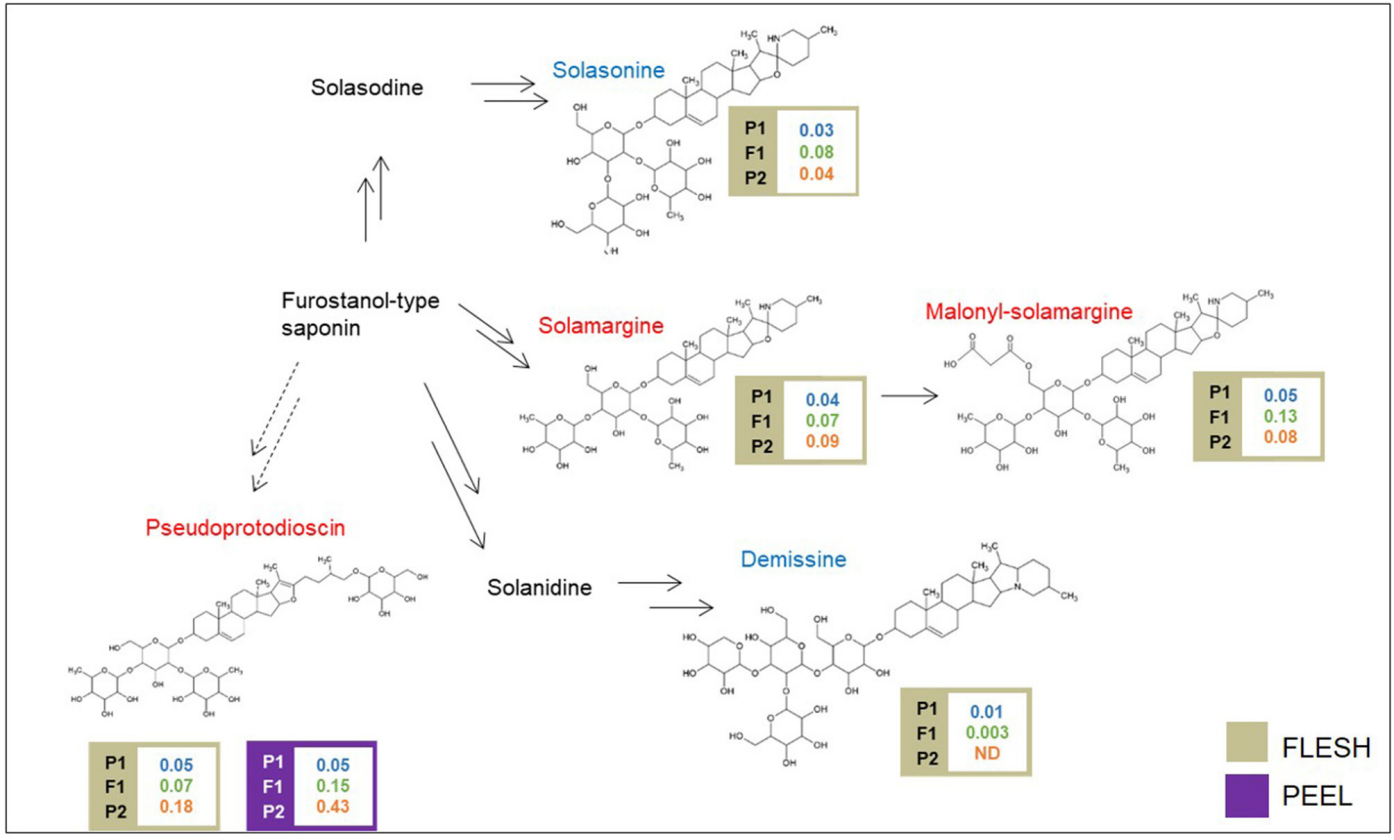
Biosynthetic pathway of steroidal glycoalkaloids. Levels of detectable metabolites are expressed as fold/ISTD (Internal Standard: formononetin) in parental lines male P1 “(67/3)”, female P2 (“305E40”), and the F1 progeny. Metabolites marked in red have been identified by the untargeted analysis, in blue by the targeted one.

A schematic biosynthetic pathway of eggplant SGAs is shown in Figure 3. Solamargine was the first compound identified as DA and showed higher relative levels in the flesh of the “305E40” parent, while the F1 hybrid showed intermediate levels, thus suggesting semidominant inheritance. Solasonine, a compound also synthesized from the same precursor of solasodine, showed comparable levels in the two parental lines and largely increased levels in the F1 hybrid, suggesting overdominance. Downstream of solamargine, we identified malonyl-solamargine (Wu et al., 2009; Lelario et al., 2019), which showed higher levels of accumulation in the F1 hybrid compared to both parental lines, again suggesting overdominance. On the other hand, pseudoprotodioscin, a steroidal saponin synthesized from the first precursor of SGAs (Cardenas et al., 2015), was detected in both the flesh and peel, was 3.6- to 8.6-fold higher in the “305E40” parent, and was shown to be recessive or semidominant in the F1 hybrid.

**Figure 4:**
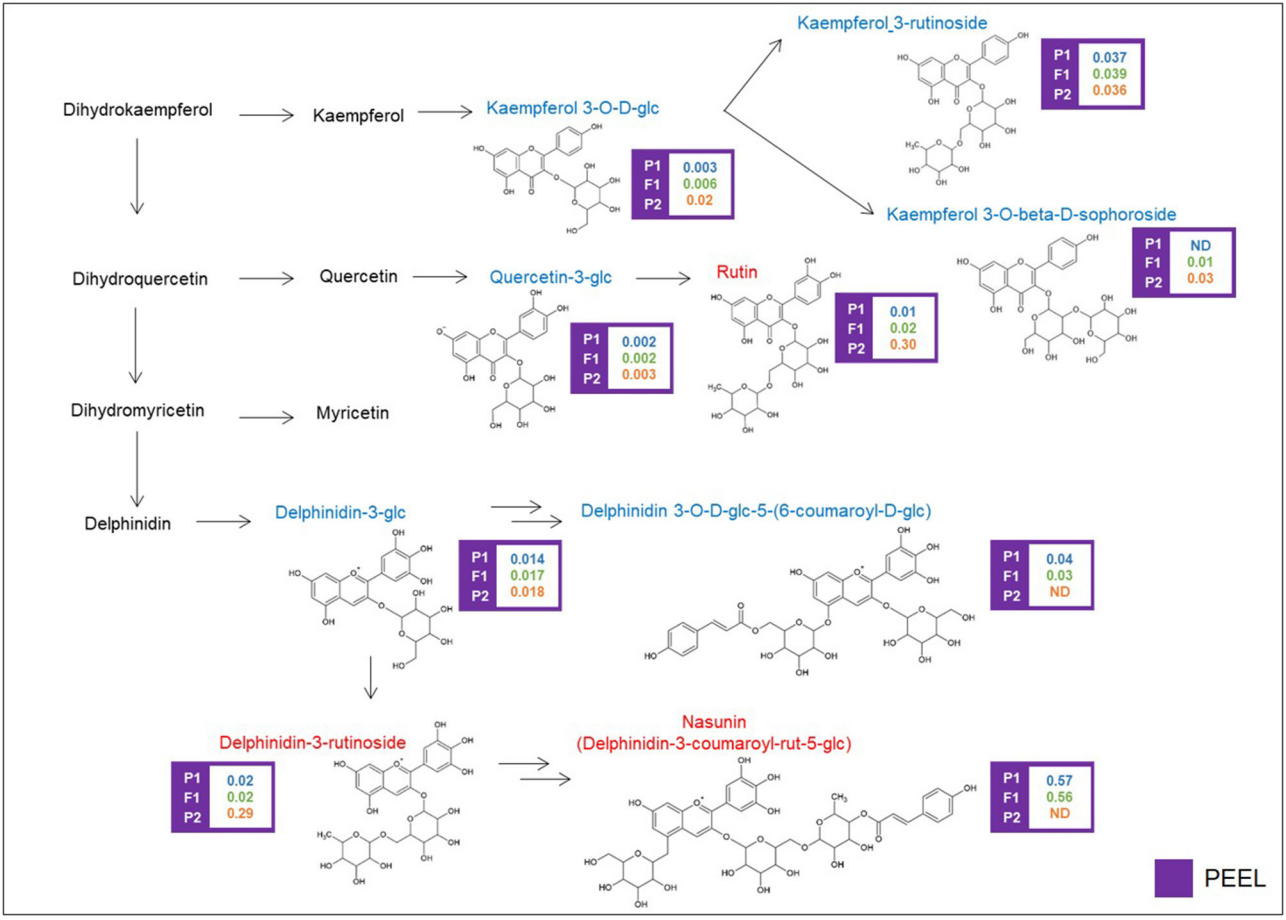
Biosynthetic pathway of flavonoids. Levels of detectable metabolites are expressed as fold/ISTD (Internal Standard: formononetin) in parental lines “67/3” (male) and “305E40” (female) and the F1 progeny. ND, not detectable. Metabolites marked in red have been identified by the untargeted analysis, in blue by the targeted one.

Glycosylated flavonols and anthocyanins were investigated in peel extracts, starting from the DA metabolites described above, and their biosynthesis is depicted in Figure 4. Rutin was 30-fold more abundant in the “305E40” parent and was recessive in the F1 hybrid. A similar trend was shown by a second compound carrying a rutinoside moiety, delphinidin-3-rutinoside (D3R). The direct precursors of these two compounds, i.e., quercetin 3-glucoside and delphinidin-3-glucoside, showed very similar relative levels in the two parental lines and in the hybrid. However, two coumaroylated derivatives of delphinidin, i.e., delphinidin 3-*O*-d-glucoside-5-(6-coumaroyl-d-glucoside) and nasunin (delphinidin-3-coumaroyl-rutinoside-5-glucoside), showed the opposite trend with respect to rutin and D3R, being more abundant in the “67/3” parent and semidominant or dominant in the F1 hybrid. This is suggestive of a clear biochemical mechanism underlying the mQTL controlling fruit pigmentation (see **Discussion**).

**Figure 5:**
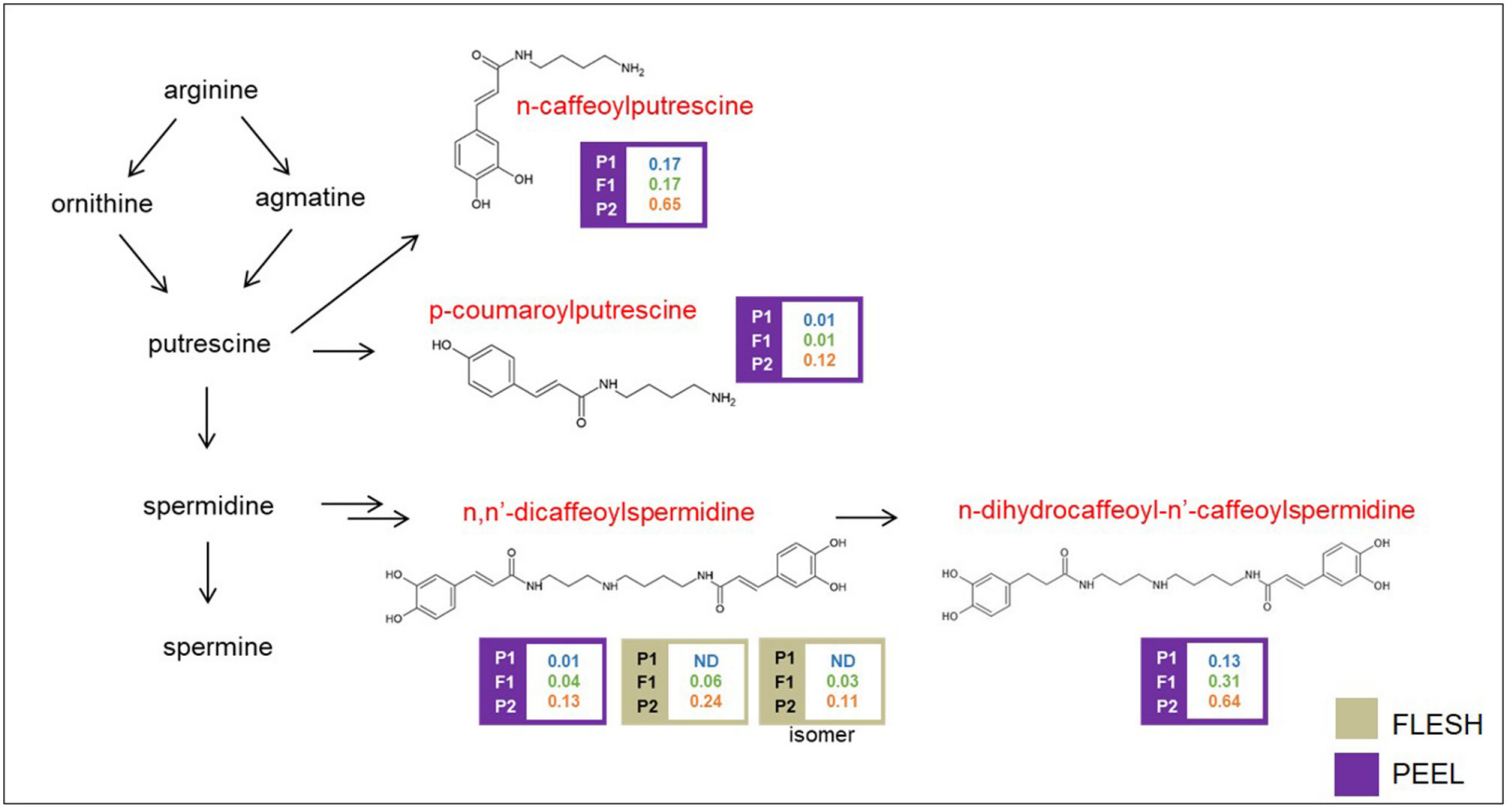
Biosynthetic pathway of polyamine conjugates. Levels of detectable metabolites are expressed as fold/ISTD (Internal Standard: formononetin) in parental lines “67/3” (male) and “305E40” (female) and the F1 progeny. Metabolites marked in red were identified by the untargeted analysis.

Polyamine conjugates are derived from the polyamines spermidine and putrescine (Figure 5). Unfortunately, our LC/MS settings did not allow the measurement of these precursors. All the polyamine conjugates we identified were strongly upregulated in the “305E40” parent and recessive or semidominant in HF1, again suggesting a possible biochemical mechanism underlying this metabolic trait (see **Discussion**).

### Targeted Metabolic Profiling of the RIL Population

**Figure 6:**
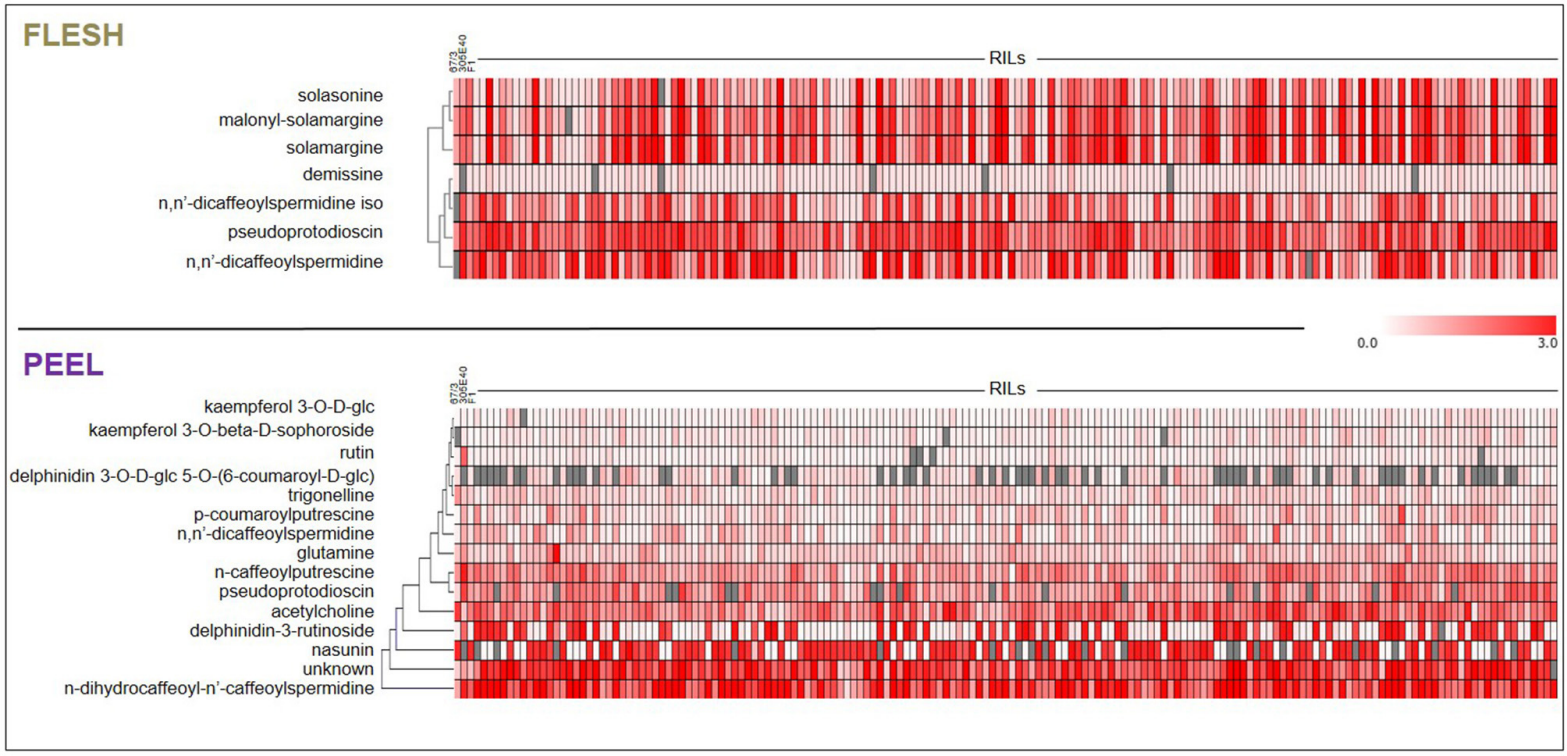
Heat-maps representing levels of accumulation of differentially metabolites in flesh and peel extracts. Metabolites were subjected to Hierarchical Clustering Analysis (HCA). Color coding represents the average signal intensity (fold/ISTD) in individual lines.

The flesh and peel fractions of the RILs were analyzed for the presence of all metabolites shown in Table 1. Two heatmaps summarizing the results are shown in Figure 6, and the detailed results are shown in Supplementary Tables 2, 3. As can be seen, related metabolites often clustered together in the heatmap, indicating a strong co-segregation in the RIL population. This can be observed, for instance, for solasonine/solamargine/malonyl-solamargine in the flesh and for kaempferol 3-*O*-glucoside/kaempferol 3-*O*-beta-d-sophoroside/rutin and delphinidin-3-rutinoside/nasunin in the peel.

### Metabolic Variation and Inter-trait Correlations

A summary of the metabolic performance for each trait in the two parental lines, the F1, and the RILs, together with broad-sense heritability ($h_{BS}^{2}$) and other parameters such as skewness, kurtosis, and presence of transgressive genotypes, is listed in Table 1 and Supplementary Table S4.

Transgressive genotypes among the RILs were identified for several metabolites in the study, generally accumulating higher relative levels than the higher accumulating parent “305E40,” with the exclusion of some compounds, such as *n*-caffeoylputrescine (P-CAPTR), for which the transgressive RILs were characterized by a reduced accumulation. Heritability was overall high, ranging from 0.48 (P-TRIG) to 0.95 (P-D3R and P-NAN).

Significant positive and negative inter-trait correlations were detected (Supplementary Figure 15). As an example, delphinidin-3-rutinoside (P-D3R) was negatively (*p* < 0.01) correlated in the peel with nasunin (P-NAN), the alternative anthocyanin form synthesized in the same tissue, and glutamine (P-GLUT), and, in the flesh, with demissine (F-DEM), malonyl-solamargine (F-MSOLM), and solasonine (F-SOLA). Conversely, delphinidin-3-rutinoside (P-D3R) was positively correlated in the peel with biochemically related metabolites such as rutin (P-RUT) and kaempferol-3-*O*-d-glucoside (P-K3G) and, moreover, with pseudoprotodioscin (P-PPROT), *n, n*′-dicaffeoylspermidine (P-DCASP), *n*-caffeoylputrescine (P-CAPTR), *n*-dihydrocaffeoyl-*n*′-caffeoylspermidine (P-DHCNCS), and *n*-*p*-coumaroylputrescine (P-COPTR), while in the flesh level it was positively correlated with both isomers of *n, n*′-dicaffeoylspermidine (F-DCASP and F-DCASP2). Conversely, nasunin (P-NAN) was negatively correlated with pseudoprotodioscin (P-PPROT), *n, n*′-dicaffeoylspermidine (P-DCASP), *n*-caffeoylputrescine (P-CAPTR), *n*-dihydrocaffeoyl-*n*′-caffeoylspermidine (P-DHCNCS), and *n*-*p*-coumaroylputrescine (P-COPTR).

### Mapping of mQTLs

**Figure 7:**
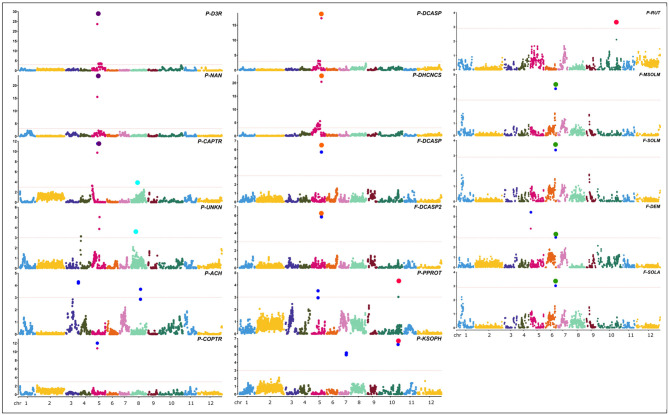
Positions of QTLs identified for the metabolites in study. Red lines in the Manhattan plots indicate LOD significance threshold. Blue dots represent significant markers within the confidence interval of the QTL (LODmax-1 interval), with LOD values plotted against genome locations. Cluster of QTLs are indicated as purple (P - FLAVO/POLY 5), orange (F/P - POLY 5), green (F-GLYKO 6), light blue (P-POLY/ ACTPH 8), and red (P-FLAVO/GLYKO 10) dots.

QTL analyses were performed using a recently developed map (Toppino et al., 2020), constituting 7,249 SNP markers, and yielded a total of 16 major QTLs (PVE > 10%) and eight minor QTLs (Table 2 and Figure 7). The largest single QTL effect was associated with P-D3R, with a PVE of 57.3%. The additive effects of all QTLs were significant at *p* < 0.05.

**Table 2:** mQTLs and mQTL clusters detected in the mapping population.

**Cluster**	**QTL**	**GW**	**Chr**.	**cM**	**Marker**	**LOD**	**CI**	**% PVE**	**A**
	*P-ACH.3.1*	3.3	03	146.8	CH03_76097038	4.29	146.8	10.7	−0.0506
	*P-UNKN.4.1*	3.1	04	8.2	CH04_3119177	3.13	8.2	6.9	−0.1002
	*F-DEM.5.1*	3.2	05	5.8	CH05_7953029	5.42	4.0–5.9	14.0	−0.0028
	*P-COPTR.5.1*	3	05	61.5	CH05_34699887	12.01	61.5–63.5	29.5	0.0332
*P-FLAVO/POLY 5*	*P-D3R.5.1*	3.4	05	66.4	CH05_36124744	28.91	64.0–66.0	57.3	0.2324
	*P-NAN.5.1*	3.3	05	66.4	CH05_36124744	23.69	64.3–66.4	54.0	−0.2202
	*P-CAPTR.5.1*	3.3	05	66.4	CH05_36124744	11.53	64.3–66.4	26.2	0.0457
	*P-PPROT.5.1*	3.2	05	75.5	CH05_37533757	3.52	75.5	9.1	0.0321
	*P-UNKN.5.1*		05	88.2	CH05_38677476	5.03	86.1–92.2	11.4	0.1325
*F/P-POLY 5*	*F-DCASP.5.1*	3.1	05	116.4	CH05_39954700	6.54	116.1–120.3	17.3	0.0529
	*F-DCASP2.5.1*	3.3	05	116.4	CH05_39954700	6.12	116.1–120.3	16.3	0.0261
	*P-DCASP.5.1*	3.2	05	116.4	CH05_39954700	18.67	116.4	42.4	0.0388
	*P-DHCNCS.5.1*	3.2	05	116.4	CH05_39954700	22.45	116.1–120.3	48.5	0.3492
*F-GLYKO 6*	*F-MSOLM.6.1*	2.7	06	122.0	CH06_98079686	4.20	122.0	11.5	−0.0696
	*F-SOLM.6.1*	2.6	06	122.0	CH06_98079686	3.69	121.3–123.1	10.1	−0.1071
	*F-SOLA.6.1*	2.7	06	122.0	CH06_98079686	3.32	121.3–123.1	9.2	−0.0497
	*F-DEM.6.1*		06	123.1	CH06_96696408	3.15	123.1	7.8	−0.0020
	*P-KSOPH.7.1*	3.2	07	86.4	CH07_131303988	5.18	86.4	11.8	0.0088
*P-POLY/UNKN 8*	*P-UNKN.8.1*		08	54.6	CH08_73641123	3.61	54.6–55.6	8.0	0.1079
	*P-CAPTR.8.1*		08	75.7	CH08_73925400	3.89	75.8	7.7	0.0245
	*P-ACH.8.1*		08	113.1	CH08_69973401	3.68	113.0	9.1	0.0506
*P-FLAVO/GLYKO 10*	*P-RUT.10.1*	3.1	10	230.2	CH10_94979390	3.34	228.2–230.2	9.4	−0.0042
	*P-KSOPH.10.1*		10	231.5	CH10_94779014	6.62	231.5	15.5	0.0099
	*P-PPROT.10.1*		10	236.0	CH10_94997738	4.32	236.9	11.3	0.0350

*For each metabolite, the chromosome, the closest marker, and the LOD value are indicated, along with the confidence interval (CI), the percentage of variation explained (%PVE), and the genome-wide thresholds (GW) at p = 0.05 (as determined from 1,000 permutations). Values in A indicate the directionality of the QTL (i.e., for positive values, the “305E40” parent contributes higher relative levels of the metabolite; for negative values, the opposite is true)*.

Twenty-four QTLs were discovered, distributed over seven chromosomes, namely, chrs. 03, 04, 05, 06, 07, 08, and 10, comprising eight isolated QTLs and five QTL clusters (Figure 7), whose position, closest marker, LOD, and confidence interval are detailed in Table 2. The isolated QTLs controlled the levels of ACH (chr. 03, 146.8 cM, and chr. 08, 113.1 cM), and UNKN (04, 8.2 cM, and 05, 88.2 cM) in the peel; DEM in the flesh (chr. 05, 5.8 cM); and COPTR (chr. 05, 61.5 cM, the only major QTL), PPROT (chr. 05, 75.5 cM, and chr. 10, 237.0 cM), and KSOPH (chr. 07, 86.4 cM, and chr. 10, 231.4 cM) in the peel.

Of the QTL clusters, one maps around 66.4 cM on chr. 05 and contains three overlapping major QTLs for delphinidin-3-rutinoside (P-D3R), nasunin (P-NAN), and *n*-caffeoylputrescine (P-CAPTR); a second QTL cluster maps around 116.4 cM on chr. 05 and includes two major QTLs for *n, n*′-dicaffeoylspermidine (F-DCASP_05) and its isomer (F-DCASP2_05), plus two major QTLs for *n, n*′-dicaffeoylspermidine (P-DCASP_05) and *n*-dihydrocaffeoyl-*n*′-caffeoylspermidine (P-DHCNCS_05); the third one maps between 122.0 and 123.1 cM on chr. 06 and includes two major QTLs for solamargine and malonyl-solamargine (F-SOLM.6.1 and F-MSOLM.6.1) and minor QTLs for demissine (F-DEM) and solasonine (F-SOLA); a fourth one maps between 54.6 and 75.8 cM on chr. 08 and contains overlapping minor QTLs for an unknown (P-UNKN.8.1) and *n*-caffeoylputrescine (P-CAPTR.8.1); and, finally, a cluster mapping on chr. 10 between 230.2 and 237.0 cM contained major QTLs for kaempferol 3-*O*-beta-d-sophoroside (P-KSOPH.10.1) and pseudoprotodioscin (P-PPROT.10.1) and a minor QTL for rutin (P-RUT.10.1).

### Candidate Genes Underlying the mQTLs

The candidate genes putatively involved in the genetic control of the metabolites in this study were identified among those underlying the confidence intervals of the identified QTLs and QTL clusters (shown in Supplementary Table 5). The possible impact of SNPs and/or indels (if any) identified in the “305E40” with respect to the “67/3” reference sequence was predicted using the SnpEff program (Cingolani et al., 2012). For each QTL or cluster of QTLs, the list of selected candidate genes is reported in Table 3, along with the annotation, the SnpEff-predicted effect on the function of the encoded protein in the “305E40” allele variant (Supplementary Table 5), and the expression in “67/3” fruits at 2–4 cm (stage 1), at commercial ripening (stage 2), and at physiological ripening (stage 3).

**Table 3:** Candidate genes underlying the mQTLs and mQTL clusters.

**Cluster/QTL**	**Genes within CI**	**Candidate Gene**	**Annotation**	**SnpEff impact**	**FPKM stage 1**	**FPKM stage 2**	**FPKM stage 3**
*P-ACH.3.1*	27	SMEL_003g182830.1	Similar to ND4: NADH-ubiquinone oxidoreductase chain 4	Mod	0.26	0.90	4.59
*F-DEM.5.1*	55	SMEL_005g229780.1	Protein of unknown function	Mod	0.00	0.48	0.00
		SMEL_005g229640.1	Similar to ycf2-A	Mod	0.00	0.00	0.00
		SMEL_005g230040.1	Similar to UGT94E5: Beta-d-glucosyl crocetin beta-1%2C6-glucosyltransferase	No	13.40	112.15	29.69
*P-COPTR.5.1*	45	SMEL_005g235160.1	Similar to At4g09670: Uncharacterized oxidoreductase At4g09670	Mod	20.59	22.49	56.48
		SMEL_005g235240.1	Similar to WRKY51: Probable WRKY transcription factor 51	Mod	0.00	0.09	0.00
		SMEL_005g235090.1	Similar to KTI2: Kunitz trypsin inhibitor 2	Mod	57.38	9.35	0.00
*P-FLAVO/POLY 5*	73	SMEL_005g235650.1	Similar to CRY1: Cryptochrome-1	No	9.53	7.77	8.58
		SMEL_005g235690.1	Similar to UGT85A24: 7-deoxyloganetin glucosyltransferase	High	0.00	0.09	0.00
		SMEL_005g235810.1	Similar to AGL70: Agamous-like MADS-box protein AGL70	High	17.46	16.39	25.61
		SMEL_005g235830.1	Protein of unknown function	High	0.00	5.05	19.96
		SMEL_005g236240.1	Similar to BEAT: Acetyl-CoA-benzyl alcohol acetyltransferase	High	34.71	50.28	0.24
*P-PPROT.5.1*	5	SMEL_005g236880.1	Protein of unknown function	Mod	6.00	19.95	10.13
		SMEL_005g236890.1	Similar to Polcalcin Cup a 4	Mod	0.00	0.46	0.00
		SMEL_005g236900.1	Similar to SYRV3: Polcalcin Syr v 3	Mod	0.00	0.00	0.00
*F/P-POLY 5*	134	SMEL_005g240620.1	Similar to At5g48380: Probably inactive leucine-rich repeat receptor-like protein kinase	High	2.73	5.28	0.63
		SMEL_005g237380.1	Protein of unknown function	High	0.00	0.00	0.00
		SMEL_005g240050.1	Similar to N: TMV resistance protein N	High	0.00	0.01	0.12
		SMEL_005g240370.1	Similar to SALAT: Salutaridinol 7-*O*-acetyltransferase	Mod	35.78	32.00	0.28
		SMEL_005g240400.1		Mod	1.89	2.93	2.88
		SMEL_005g240350.1	Similar to BEAT: Acetyl-CoA-benzyl alcohol acetyltransferase	Mod	1.40	0.20	0.00
		SMEL_005g240550.1		Mod	100.36	69.23	0.07
*F-GLYKO 6*	159	SMEL_006g259360.1	Similar to N: TMV resistance protein N	Mod	2.45	2.56	2.03
		SMEL_006g258510.1	Similar to ndhH: NAD(P)H-quinone oxidoreductase subunit H%2C chloroplastic	Mod	0.00	0.00	0.00
		SMEL_006g258660.1	Similar to DTX54: Protein DETOXIFICATION 54	Mod	0.00	0.00	0.00
		SMEL_006g258670.1		Mod	0.00	0.00	0.00
		SMEL_006g259540.1	Similar to CYP71D7: Cytochrome P450 71D7	No	0.00	0.00	0.00
		SMEL_006g259050.1	Similar to CYP716A15: Beta-amyrin 28-monooxygenase	No	0.00	0.00	0.00
		SMEL_006g259060.1		No	0.48	0.42	0.00
*P-KSOPH.7.1*	34	SMEL_007g288740.1	Similar to CYCU1-1: Cyclin-U1-1	Mod	0.70	0.17	0.00
		SMEL_007g288700.1	Protein of unknown function	Mod	19.47	9.53	9.66
		SMEL_007g288720.1	Similar to PCMP-A3: Pentatricopeptide repeat-containing protein At1g71460%2C	Mod	2.00	1.73	2.81
		SMEL_007g288660.1	Similar to PER3: Peroxidase 3 (*Arabidopsis thaliana* OX%3D3702)	Mod	62.19	23.87	17.71
		SMEL_007g288690.1		Mod	0.00	0.00	0.08
		SMEL_007g288730.1	Similar to PEX22: Peroxisome biogenesis protein 22	Mod	21.72	23.65	41.05
		SMEL_007g288920.1	Similar to BHLH117: Transcription factor bHLH117	No	0.00	0.00	0.00
*P-POLY/UNKN 8*	26	SMEL_008g307770.1	Similar to PCMP-E22: Pentatricopeptide repeat-containing protein At2g02750	High	0.00	0.00	0.00
		SMEL_008g307900.1	Similar to ycf2-A: Protein Ycf2	High	0.00	0.00	0.00
		SMEL_008g307850.1	Protein of unknown function	High	0.00	0.00	0.00
		SMEL_008g307910.1	Similar to PAB7: Polyadenylate-binding protein 7	High	0.00	0.00	0.00
		SMEL_008g307740.1	Similar to CBL10: Calcineurin B-like protein 10	High	12.01	9.91	4.77
		SMEL_008g307920.1	Similar to Acyl-lipid (9-3)-desaturase	Mod	22.21	14.70	22.65
*P-ACH.8.1*	10	SMEL_008g307500.1	Protein of unknown function	Mod	0.00	0.00	0.00
		SMEL_008g307450.1	Similar to vacuolar-processing enzyme	Mod	1.32	2.30	0.09
		SMEL_008g307530.1	Similar to At4g32950: Probable protein phosphatase 2C 61	Mod	0.00	0.06	0.15
		SMEL_008g307440.1	Similar to vacuolar-processing enzyme	Mod	107.04	89.38	428.99
		SMEL_008g307520.1	Similar to At4g32950: Probable protein phosphatase 2C 61	Mod	0.00	0.00	0.07
*P-FLAVO/GLYKO 10*	40	SMEL_010g353210.1	Protein of unknown function	High	0.00	0.00	0.00
		SMEL_010g352840.1	Similar to WAK2: Wall-associated receptor kinase 2	Mod	0.00	0.00	0.00
		SMEL_010g352880.1	Similar to PYL4: Abscisic acid receptor PYL4	No	0.16	0.08	0.00
		SMEL_010g352930.1	Similar to DREB2C: Dehydration-responsive element-binding protein 2C	No	0.00	0.00	0.00
		SMEL_010g353090.1	Similar to RAPTOR1: Regulatory-associated protein of TOR 1	No	7.28	4.16	7.11
		SMEL_010g353110.1		No	0.32	1.43	1.33
		SMEL_010g353170.1	Similar to peroxidases and cationic peroxidases	No	0.00	0.00	0.00
		SMEL_010g353190.1		No	0.00	0.11	0.15

*For each mQTL/mQTL cluster, the total number of genes within the confidence interval, the candidate genes, and the impact of the mutations (high, moderate, or no effect) is indicated. SnpEff output for the below candidate genes is reported in Supplementary Table 5. Expression values (FPKM, from Barchi et al., 2019) for candidate genes in fruits at 2–4 cm (stage 1), at commercial ripening (stage 2), and at physiological ripening (stage 3) of the line “67/3” are reported*.

## Discussion

### mQTLs Mapping

Genome-wide metabolic profiling is a powerful tool to identify components of fruit metabolic composition and isolate the underlying genes. Metabolic profiling of RIL and/or introgression line (IL) populations was used to map metabolic QTLs controlling biomass production content in *Arabidopsis* (Lisec et al., 2008) or influencing nutritional quality in tomato fruits (Schauer et al., 2006) and potato tubers (Carreno-Quintero et al., 2012). With respect to other Solanaceae, eggplant genome-wide metabolic profiling has lagged behind, mainly due to the lack, until recently, of a high-quality reference sequence and of reliable metabolic profiling methods. A chromosome-anchored eggplant genome sequence has been recently developed (Barchi et al., 2019) and, in combination with the metabolic profiling methods described here, paves the way for the discovery of genes controlling major metabolic traits. Here, we describe a method for comprehensive metabolic profiling of polar metabolite eggplant fruits, based on LC/HRMS. An untargeted approach was applied to identify metabolites showing contrasting regulations in the two parental lines and the F1 hybrid. The DA metabolites, as well as additional ones biosynthetically related to those initially identified, were then quantified in an F6 population composed of 164 RILs, and the data were used to infer and map mQTLs influencing fruit nutritional quality.

Based on metabolite relative levels of the F1 hybrid and the two parental genotypes, we were able to identify overdominant, dominant, semidominant, recessive, or under-recessive traits. This was in good agreement with the transgressive genotypes identified in the RIL population, in which some individuals showed higher relative levels of metabolites with respect to the highest scoring parent “305E40” and the F1 for the dominant/overdominant metabolites malonyl-solamargine and *n, n*′-dicaffeoylspermidine and lower relative levels of the recessive metabolites delphinidin-3-rutinoside and rutin.

Major mQTLs were identified influencing the content of several classes of metabolites: SGAs (flesh and peel), anthocyanins and glycosylated flavonols (peel), polyamine conjugates (flesh and peel), and acetylcholine (peel). Analyses using a high-density genetic linkage map (Toppino et al., 2020) revealed that eight isolated QTLs and five QTL clusters control the accumulation of metabolites in this study.

### Steroidal Glycoalkaloids (SGAs)

SGAs are triterpene-derived, toxic defense compounds synthesized by many Solanaceae and Liliaceae (Cardenas et al., 2015). The main eggplant SGAs are alpha-solasonine and alpha-solamargine (Sanchez-Mata et al., 2010; Mennella et al., 2012) (Figure 3). Depending on the dosage, SGAs, including eggplant SGAs, have a variety of harmful (toxic) and beneficial (anticarcinogenic) effects in both animals and humans (Friedman, 2006, 2015). Previous studies showed that chrs. 07 and 12 of tomato, potato, and eggplant harbor clusters of genes involved in SGA biosynthesis (Itkin et al., 2013; Barchi et al., 2019).

With the untargeted approach, the SGAs solamargine and its derivative malonyl-solamargine, as well as a steroidal saponin, pseudoprotodioscin, were detected as DAs, with higher relative levels in the “305E40” parent, which carries introgressions from *S. aethiopicum* (Barchi et al., 2010), an edible species known to contain high levels of solamargine (Sanchez-Mata et al., 2010). Two additional SGAs, solasonine and demissine, were detected by pathway walking. One QTL cluster controlling the levels of solasonine, solamargine, malonyl-solamargine, and demissine maps on chr. 06 and co-localizes with the SGA QTL mapped by Toppino et al. (2016). Candidate genes underlying this cluster include two multidrug and toxin efflux (MATE) transporters, possibly involved in vacuolar sequestration of SGAs; a cytochrome P450 contributing to SGA biosynthesis in potato (Manrique-Carpintero et al., 2014); and a cluster of genes encoding possible beta-amyrin 28-monooxygenases, involved in a competing branch of triterpenoid biosynthesis (Daniel et al., 2017). A QTL for demissine in the flesh (F-DEM.5.1) contains a gene encoding a UGT94 enzyme (a tomato GAME18 homolog) (Cárdenas et al., 2019). Within the confidence interval of the major QTL on chr. 10 for pseudoprotodioscin in the peel, the gene SMEL_010g352880, coding for an abscisic acid receptor PYL4 involved in the regulation of tobacco alkaloid biosynthesis (Lackman et al., 2011), was detected. None of the SGA QTLs maps on chr. 07 or 12, which have been previously shown to harbor clusters of genes involved in SGA biosynthesis in tomato, potato, and eggplant (Itkin et al., 2013; Barchi et al., 2019), suggesting that SGA accumulation in the present population is controlled by different genomic regions. The results presented here increase our understanding of SGA inheritance and accumulation in eggplant, generating useful knowledge for the marker-assisted breeding of these bioactive compounds.

### Flavonoids

Anthocyanins and glycosylated flavonols are phenylpropanoid pigments, important both for fruit antioxidant properties and for their health-promoting effects (He and Giusti, 2010). In particular, nasunin has been described for its antioxidant and cholesterol-lowering properties (Kayamori and Igarashi, 1994; Noda et al., 2000; Casati et al., 2016). In eggplant, they are synthesized in leaves and in the peel of ripe fruits, which show large variation with regard to pigmentation (Cericola et al., 2013) (Figure 4). The genetic control of anthocyanin formation, distribution, and accumulation has been widely studied in Solanaceae species including eggplant (van Eck et al., 1993; Chaim et al., 2003; Borovsky et al., 2004; Bovy et al., 2007; Gonzali et al., 2009; Stommel and Dumm, 2015).

An F2 population derived from the same cross (“305E40” × “67/3”) was employed for the development of a RAD-tag-based linkage map and for the identification of QTL associated with phenotypic and biochemical traits, as well as for the identification of chromosome regions involved in anthocyanin distribution in eggplant tissues and organs, highlighting their synteny with tomato (Barchi et al., 2012; Ge et al., 2013; Cericola et al., 2014; Toppino et al., 2016). More recently, the RIL population employed in this study was used to elucidate the genetic basis of seven traits related to anthocyanin content in different organs (Toppino et al., 2020). Both studies revealed the presence of two main QTL clusters on chrs. 05 and 10, with the cluster on chr. 10 being mainly involved in anthocyanin intensity in leaves, stems, and corollas, while the cluster on chr. 05 is more associated with the type of anthocyanin accumulated (Barchi et al., 2012; Toppino et al., 2020). We show here that the cluster identified on chr. 10 controls the levels of the glycosylated flavonols rutin (P-RUT) and kaempferol 3-*O*-beta-d-sophoroside (P-KSOPH) in the peel. This cluster contains several candidate genes, including a PYL4 abscisic acid receptor (SMEL_010g352880), which was also identified as a candidate for P-PPROT. Indeed, abscisic acid not only seems to regulate alkaloid accumulation (see the paragraph on steroidal glycoalkaloids) but is also known to positively regulate flavonoid/anthocyanin biosynthesis in several plant species (Hattori et al., 1992; Ban et al., 2003; Diretto et al., 2020).

We identified a dehydration-responsive element-binding protein 2C and a PPC6-1—protein phosphatase 2C—lying within the cluster. Several peroxidase-encoding genes have been also identified, which may be involved in the degradation of anthocyanin, leading to pigment concentration reduction and color fading (Luo et al., 2019).

Three peroxidases have also been identified in the confidence interval of the QTL for P-KSOPH on chr. 07, together with a BHLH transcription factor, similar to one putatively involved in anthocyanin biosynthesis in peony (Zhang et al., 2015), which carries a moderate effect according to SnpEff analysis.

The cluster on chr. 05 around 66.5 cM presents different characteristics: it controls, in an opposite way, the levels of nasunin and delphinidin-3-rutinoside (D3R), the two alternative anthocyanin forms in the parental lines of the RIL population. This cluster was already described as a genomic region involved in the control of several anthocyanin-related traits, such as stem anthocyanin, corolla color, and peel fruit color (Barchi et al., 2012; Toppino et al., 2016, 2020).

Nasunin is a coumaroylated form of D3R (Figure 4), and the two compounds show opposite trends, with nasunin being higher in the “67/3” parent and dominant, while D3R is higher in the “305E40” parent and recessive. This suggests that the QTL affects the function of an acyl transferase, which is active in the “67/3” parent and totally or partially inactive in the “305E40” one. Indeed, the candidate genes underlying the QTL cluster contain the presence of a gene (SMEL_005g240420.1) with high similarity to the *Clarkia breweri* acetyl-CoA benzyl alcohol acetyltransferase (BEAT), an enzyme involved in the generation of floral scent (Dudareva et al., 1998). It is conceivable that in eggplant, this gene acts on anthocyanins instead rather than on benzyl alcohol and accepts different acyl group donors, such as coumaroyl-CoA. Analysis of additional compounds in the same pathway supports this hypothesis: delphinidin 3-glucoside-5-coumaroyl-glucoside shows distribution and dominance relationships similar to those of nasunin. The SnpEff analysis performed on the two parental lines revealed a SNP which could determine a severe effect of loss-of-function mutation in the SMEL_005g240420.1 CDS sequence of the “305E40” parent, in agreement with the biochemical data. For these reasons, a more detailed functional study of this gene was started (Toppino et al., unpublished). Other interesting genes lying within the confidence interval of the cluster are SMEL_005g235650.1, a cryptochrome 1 blue light photoreceptor, controlling photomorphogenesis and anthocyanin accumulation in tomato (Ninu et al., 1999).

### Polyamine Conjugates

Polyamines play different key functions in the regulation of many physiological processes involving floral and fruit development and leaf senescence, as well as abiotic and biotic plant stress responses (Alcázar et al., 2010; Tiburcio et al., 2014). Their schematic biosynthesis is shown in Figure 5. They are found often as conjugates with acyl (coumaroyl, caffeoyl, and sinapoyl) groups, which play a role in the defense against herbivores (Kaur et al., 2010).

We found several major mQTLs and QTL clusters for polyamine conjugates. A major cluster maps on chr. 05 around 116.4 cM, with the “305E40” parent contributing higher relative levels of these metabolites. Underlying genes comprise genes for pathogen resistance (LRR, N) and a cluster of seven acyltransferases showing similarity to BEAT as well as to salutaridinol 7-*O*-acetyltransferase (SALAT), an enzyme involved in alkaloid biosynthesis in *Papaver somniferum* (Grothe et al., 2001). All these genes show lesions with high or moderate impact in their coding sequence in one of the two parental lines.

A second cluster for polyamine conjugates maps on chr. 05 around 66.4 cM. It controls the levels of *n, n*′-dicaffeoylspermidine in the flesh and peel and (only in the peel) of *n*-dihydrocaffeoyl-*n*′-caffeoylspermidine and *n*-caffeoylputrescine. This cluster overlaps with the one controlling nasunin/D3R. However, the relative levels and dominance characteristics of polyamine conjugates are antiparallel with respect to those of nasunin. Therefore, it is difficult to assume the two overlapping QTLs are due to a single acyltransferase with broad substrate specificity, acting on both classes of compounds. Since a second acyltransferase is not found within the confidence interval of this QTL cluster, it is possible that the polyamine QTL is mediated by the action of regulatory proteins, many of which are found in this interval. Within the confidence interval of the mQTL cluster on chr. 08, which contains overlapping QTLs for P-COPTR and UNKN, five genes having a severe SnpEff impact were detected, including an acyl-lipid (9-3)-desaturase, reported to play a role in polyamine biosynthesis (Christopher and Holtum, 2000) (Supplementary Table 5).

## Conclusions

The plant metabolome represents a bridge between the genome and the phenome of plants, and thanks to the available high-throughput metabolic profiling and genotyping technologies, it is now possible to shed light on the genetic bases of metabolism in crop plants.

We previously developed a high-density linkage map by genotyping a RIL mapping population obtained from the cross of two parental lines differing for many traits, including the shape, dimension, and pigmentation of their fruits.

Our metabolomic strategy led to the identification of DA metabolites in the fruit flesh and peel, most of which belong to the glycoalkaloid, anthocyanin, and polyamine conjugate classes. The metabolic profiling of the F1 hybrid and each RIL progeny made it possible to detect for the first time mQTLs and QTL clusters influencing the regulation of these compounds and influencing the fruit nutritional quality in eggplant.

Genome annotation in combination with sequence differences in the two parental lines supplied a key tool to gather valuable information for QTL fine mapping, candidate gene identification, and the identification of molecular markers suitable for identifying favorable alleles, thus increasing the precision and efficiency of selection in breeding. Our data also demonstrate the benefits of the methods described here for broad metabolomics studies in fruit-bearing crop species.

## Data Availability Statement

For genotypic data refer to Toppino et al. (2020): https://doi.org/10.3390/genes11070745. The metabolite levels are provided in the Supplementary Tables S2, S3. The raw metabolomic data are available upon request.

## Author Contributions

MS and LB produced and analyzed the data and wrote the manuscript. LT provided the materials, analyzed the data, and wrote the manuscript. GD helped in producing the data. TS provided the materials. SL revised the manuscript. GLR conceived the study and reviewed the paper. GG conceived and coordinated the study, analyzed the data, and wrote the manuscript. All authors read and approved the final manuscript.

## Conflict of Interest

The authors declare that the research was conducted in the absence of any commercial or financial relationships that could be construed as a potential conflict of interest.

## Footnotes

^1^Available online at: http://faostat.fao.org

^2^Available online at: https://github.com/YinLiLin/CMplot
