# Supplementary material for: An Eggplant Recombinant Inbred Population Allows the Discovery of Metabolic QTLs Controlling Fruit Nutritional Quality
Source: Front Plant Sci. 2021 May 17;12:638195. doi: 10.3389/fpls.2021.638195 (PMC8166230; doi:10.3389/fpls.2021.638195)
Supplement: Supplementary file 1 [file Presentation_1.pdf]

Supplementary Text and Figures

Metabolite identification

Pseudoprotodioscin, a furostanol derived compound recently described in *S. melongena* roots (Liu et al., 2020), was detected as a 1031.5418 m/z peak detected in full MS (adduct [M+H]+, RT 12.2). By comparing the MS2 fragmentation pattern with the one produced *in silico*, we observed 4/4 fragment matches: at m/z 869.48 [M+H-1Rha]+, m/z 723.43 [M+H-2Rha]+, m/z 577.37 [M+H-2Rha-1Glc]+, and m/z 415.32 [aglycone+H]+ (Supplementary Figure S1). The measured 868.5043 m/z (RT 10.3), identified as solamargine [M+H]+, showed MS2 fragments at m/z 850.49 and 704.43 corresponding to [M+H-H20]+ and [M+H–1Rha]+, respectively. Already described fragments for solamargine at m/z 576.38 [M+H–2Rha]+, m/z 396.32 [M+H–2Rha–Glc–H_2O]+ and m/z 253.19 [$C_{19}H_{25}$+H]+ were also detected (Lelario et al., 2019) (Supplementary Figure S2). The ion m/z 954.5053 identified as malonyl-solamargine [M+H]+ (RT 11.7), showed already described fragments [M+H-CO_2]+ at m/z 910.51, [M+H–CO_2–H_2O]+ at m/z 892.50, [M+H–CO_2–Rha]+ at m/z 764.46, and at m/z 722.45 [M+H–$C_3H_2O_3$–Rha]+ (Lelario et al., 2019). In addition we observed [M+H–$C_3H_2O_3$–2Rha]+ at m/z 576.39, and the protonated solasodine aglycone at m/z 414.34, [aglycone+H-H_2O]+ at m/z 396.32, and [aglycone+H-2H_2O]+ at m/z 378.31 (Supplementary Figure S3). In flesh, two n-dicaffeoylspermidine isomers were tentatively identified at RT 1.2 at 6.8, based on their accurate mass in full MS (m/z 470.2291 and 470.2286 [M+H]+) and isotopic patterns.

Rutin and delphinidin-3-rutinoside were identified by comparing the MS2 fragmentation patterns with those of authentic standards (Supplementary Figure S4-S5). Nasunin, also known as delphinidin-3-(p-coumaroyl)-rutinoside-5-glucoside, was detected as M+ (919.2522 m/z, RT 8.1), and MS2 fragments were at m/z 757.13 corresponding to [M-Glc]+, m/z 465.10 [M-p-coumaroyl-Glc]+, and m/z 303.07 [delphinidin aglycone]+, as already described by (Ichiyanagi et al., 2005) (Supplementary Figure S6). Within the polyamine conjugates we tentatively identified in peel, the ion m/z 251.1388 (RT 3.1) identified as n-caffeoylputrescine [M+H]+, showed MS2 fragments m/z 163.06 and m/z 89.16, which are characteristic for the caffeoyl- and putrescine [amine+H]+ moieties (Baumert et al., 2001); in addition, we observed a signal at m/z 234.11 generated by the loss of NH_3 (Supplementary Figure S7). Glutamine (m/z 147.0763, RT 1.2) and acetylcholine (m/z 146.1175, RT 1.1), detected as [M+H]+ and [M]+ adducts, respectively, were confirmed by authentic standard (Supplementary Figure S8-S9). The ion m/z 138.0549 (RT 1.1) was tentatively identified as alkaloid trigonelline, by MS2 fragments showing, together with [M+H-H_2O]+ at m/z 121.01, the [M+H-COOH]+ at m/z 94.10 generated by the pyridine ring carrying the methyl-group, excluding other putative IDs with the same $C_7H_7NO_2$ formula (e.g. anthranilic acid, methyl nicotinic acid, aminobenzoic acid, pyridylacetic acid). MS2 signals at m/z 94.10 and 138.10 deriving from trigonelline were previously described in foxtail millet (Li et al., 2018). Solasonine identification (m/z 884.5001, RT 10.2) was confirmed by the MS2 fragmentation pattern showing, in addition to the [M+H-H_2O]+ adduct at m/z 866.49, loss of rhamnose at m/z 738.44, loss of glucose at m/z 704.44, and, as previously described for malonyl-solamargine, the protonated solasodine aglycone at m/z 414.34, [aglycone+H-H_2O]+ at m/z 396.32, and [aglycone+H-2H_2O]+ at m/z 378.31 (Supplementary Figure S11). In peel, we identified kaempferol-3-glucoside (m/z 449.1079, RT 10.1) by comparison with authentic standard (Supplementary Figure S12); kaempferol 3-O-beta-D-sophoroside (m/z 611.1611 corresponding to the [M+H]+ adduct (RT 8.6)) was tentatively identified by the MS2 fragments at m/z 287.06 given by [aglycone+H]+, at m/z 449.10 due to loss of glucose, and at m/z 431.10 due to loss of glucose and H_2O (Supplementary Figure S13). The anthocyanin delphinidin 3-O-D-glucoside-5-(6-coumaroyl-D-glucoside) (m/z 773.1935, RT 11.0), showed 5 out of 5 MS2 fragments matching the *in silico* fragmentation performed with Metfrag (Supplementary Figure S14).

References

Baumert, A., Mock, H.-P., Schmidt, J., Herbers, K., Sonnewald, U., and Strack, D. (2001). Patterns of phenylpropanoids in non-inoculated and potato virus Y-inoculated leaves of transgenic tobacco plants expressing yeast-derived invertase. *Phytochemistry* 56(6)**,** 535-541.

Ichiyanagi, T., Kashiwada, Y., Shida, Y., Ikeshiro, Y., Kaneyuki, T., and Konishi, T. (2005). Nasunin from Eggplant Consists of Cis− Trans Isomers of Delphinidin 3-[4-(p-Coumaroyl)-l-rhamnosyl (1→ 6) glucopyranoside]-5-glucopyranoside. *Journal of agricultural and food chemistry* 53(24)**,** 9472-9477.

Lelario, F., De Maria, S., Rivelli, A.R., Russo, D., Milella, L., Bufo, S.A., et al. (2019). A complete survey of glycoalkaloids using LC-FTICR-MS and IRMPD in a commercial variety and a local landrace of eggplant (Solanum melongena L.) and their anticholinesterase and antioxidant activities. *Toxins* 11(4)**,** 230.

Li, S., Dong, X., Fan, G., Yang, Q., Shi, J., Wei, W., et al. (2018). Comprehensive profiling and inheritance patterns of metabolites in foxtail millet. *Frontiers in plant science* 9**,** 1716.

Liu, Y., Yin, X., Sun, Y.-p., Liu, Y., Zhou, Y.-Y., Pan, J., et al. (2020). Chemical constituent from the roots of Solanum melongena L. and their potential anti-inflammatory activity. *Natural Product Research***,** 1-8.

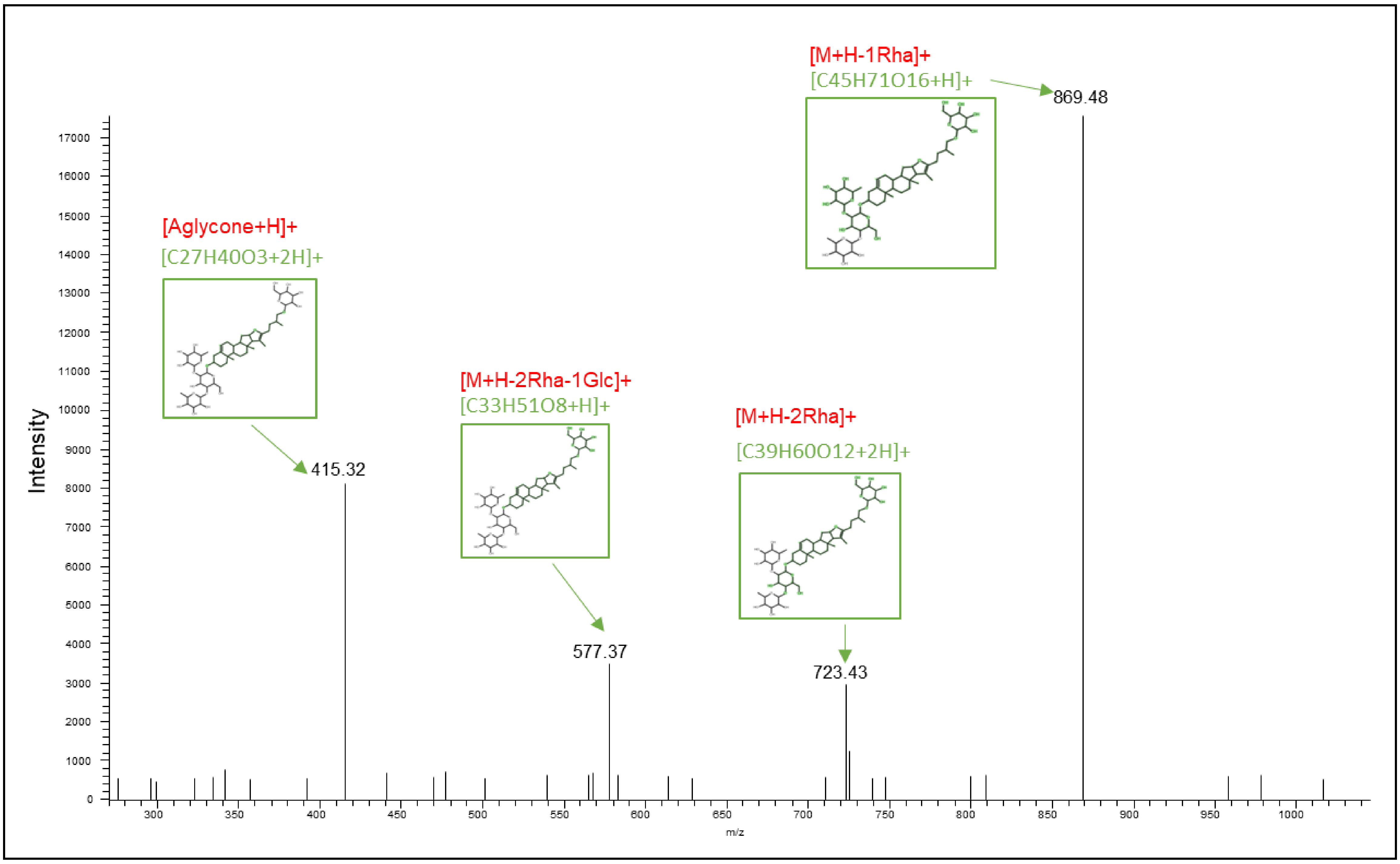

Supplementary Figure S1. MS^2 fragmentation pattern showing the most abundant signals produced by the 1031.5418 m/z parent ion (RT: 12.2), identified as pseudoprotodioscin (M+H)+. Green squares represent the MS^2 fragments of the parent compound predicted by MetFrag.

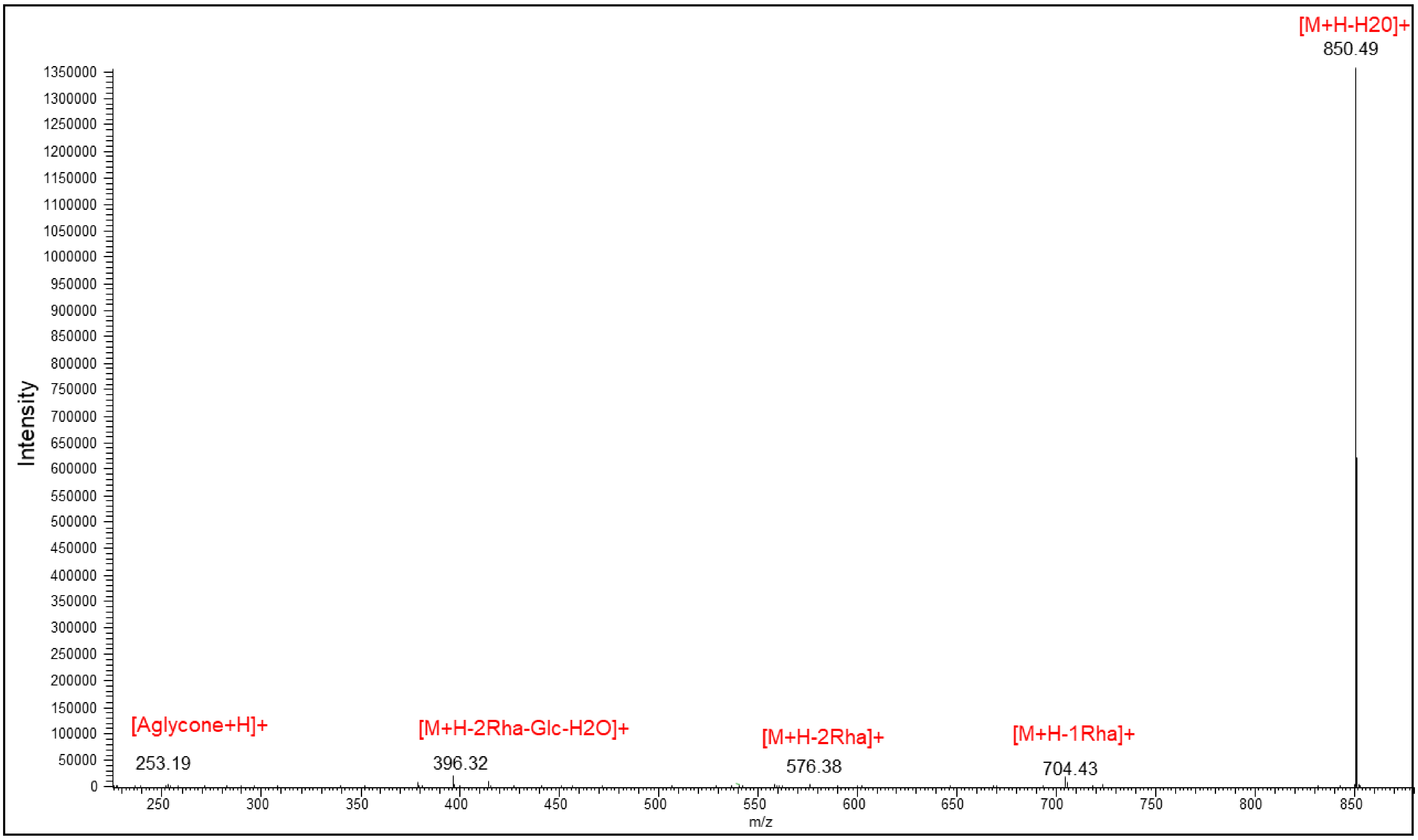

Supplementary Figure S2. MS^2 fragmentation pattern showing the most abundant ions produced by the 868.5043 m/z precursor (RT 10.3), identified as solamargine (M+H)+.

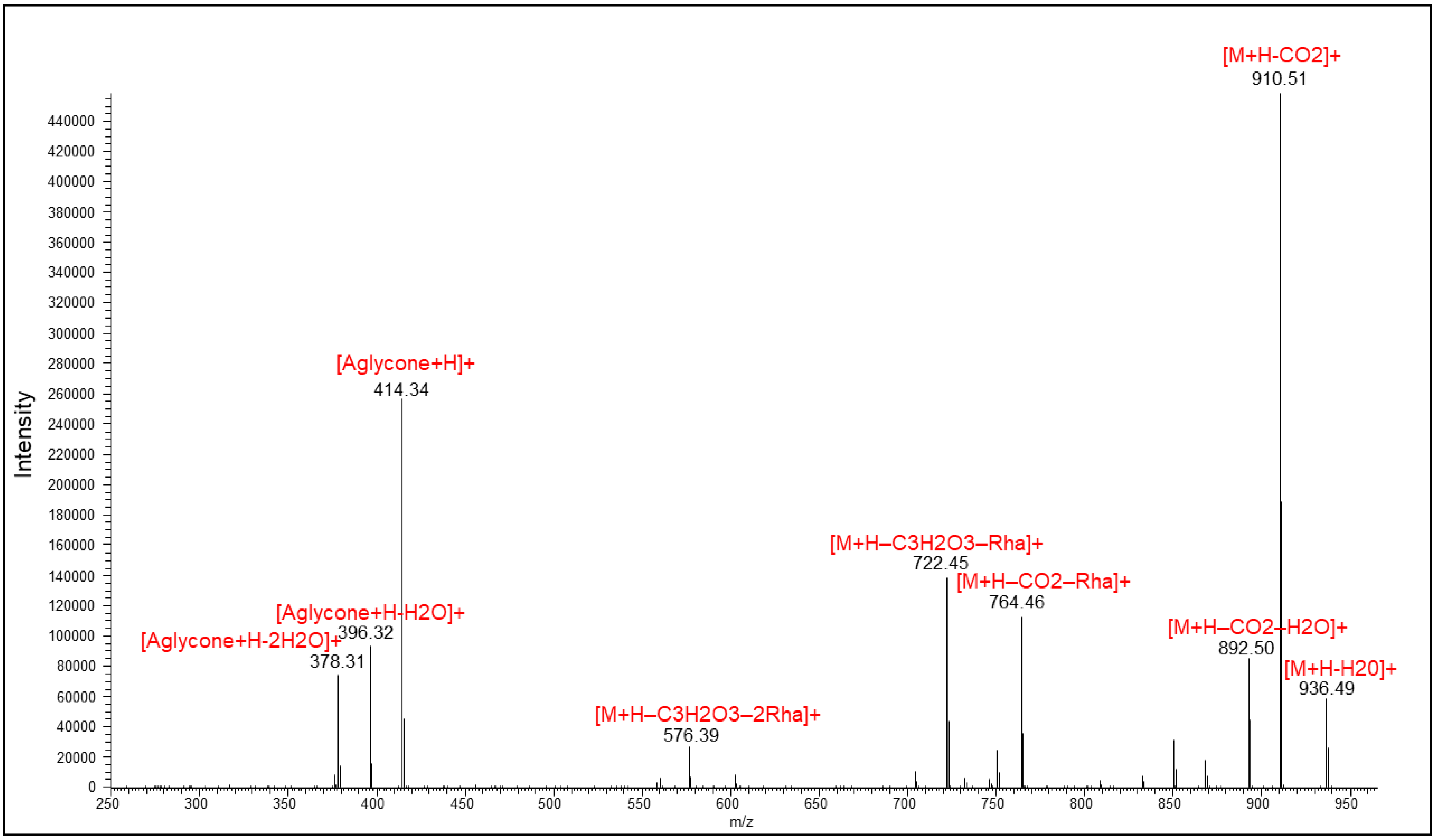

Supplementary Figure S3. MS^2 fragmentation pattern showing the most abundant ions produced by 954.5053 m/z precursor (RT 11.7), identified as malonyl-solamargine (M+H)+.

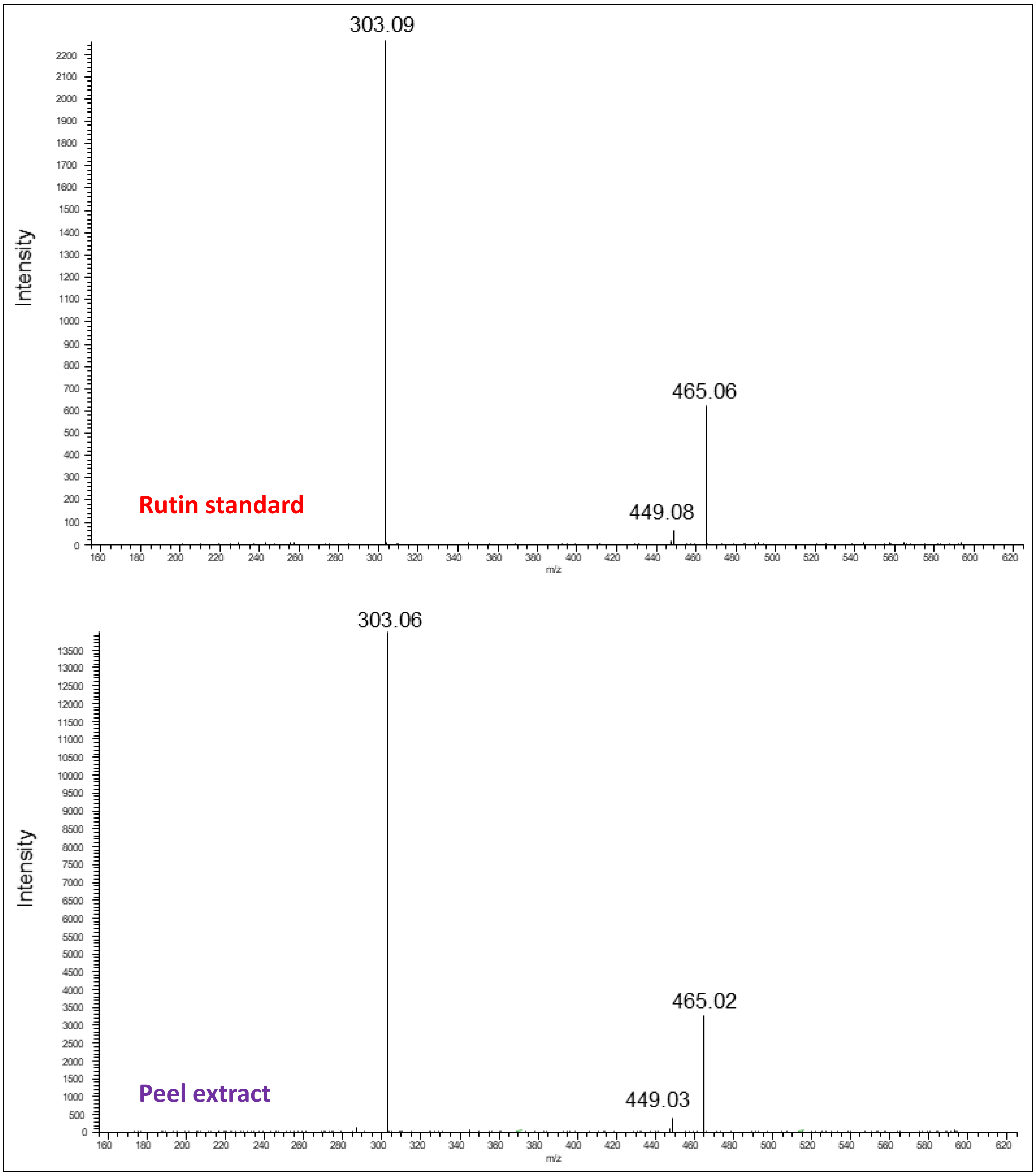

Supplementary Figure S4. Confirmation of the identity of the ion 611. 1610 m/z (RT 8.9) by comparison of MS2 signals generated by parental ion in rutin authentic standard and peel extract.

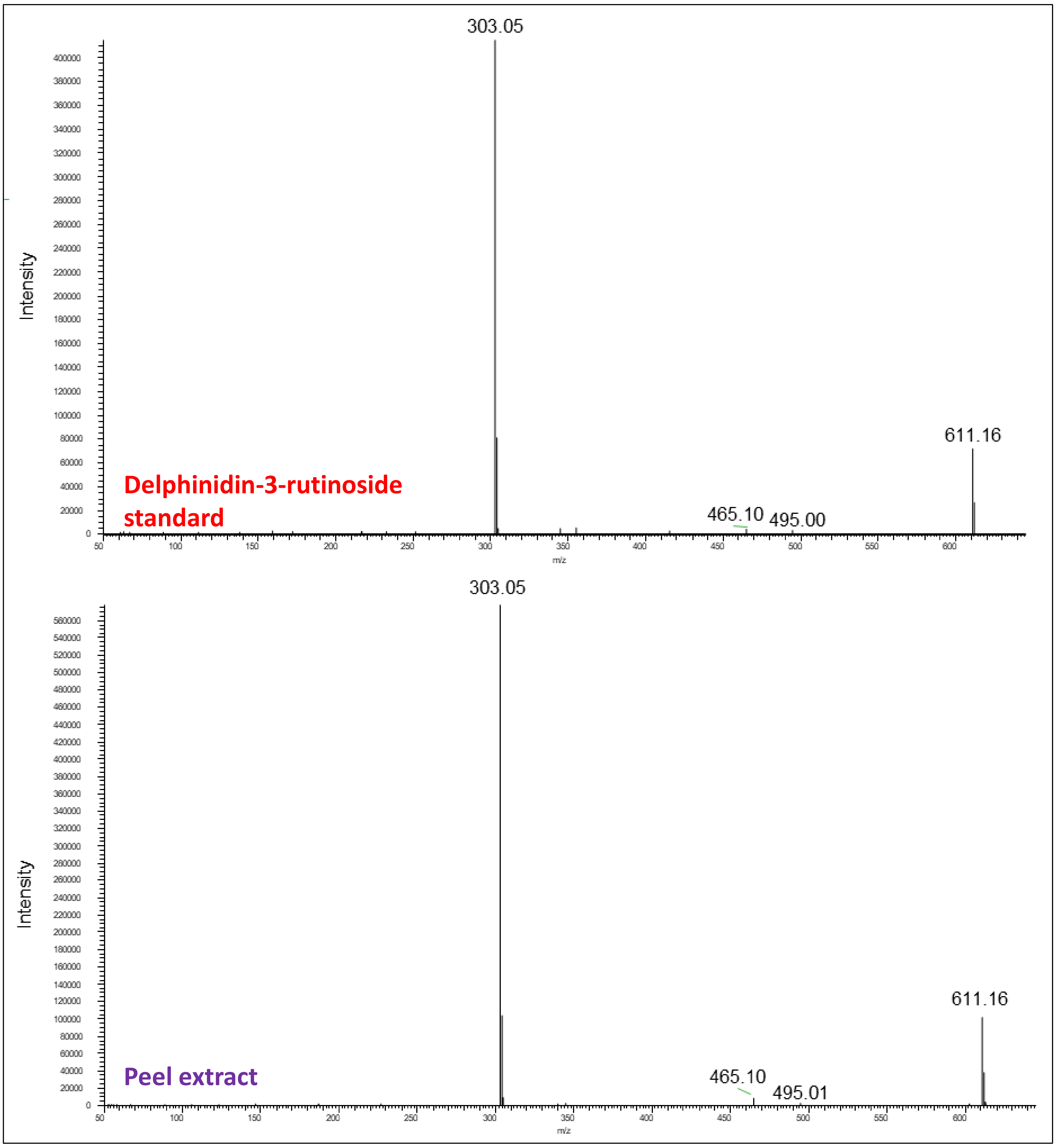

Supplementary Figure S5. Confirmation of the identity of the ion 611. 1609 m/z (RT 5.6) by comparison of MS2 signals generated by parental ion in delphinidin-3-rutinoside authentic standard and peel extract.

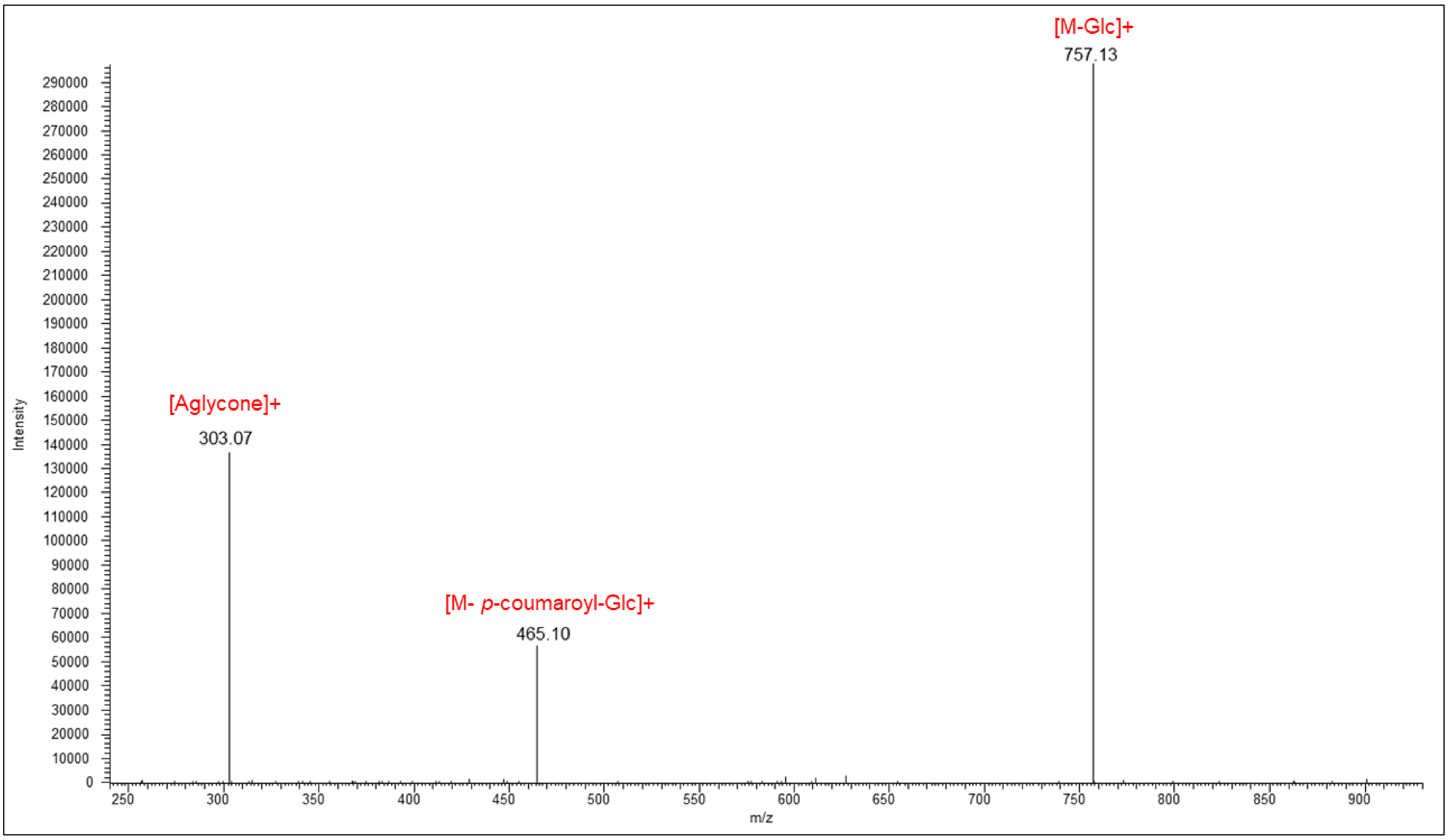

Supplementary Figure S6. Detected MS^2 fragmentation pattern showing the most abundant ions produced by 919.2522 m/z precursor (RT 8.1), identified as nasunin (M+).

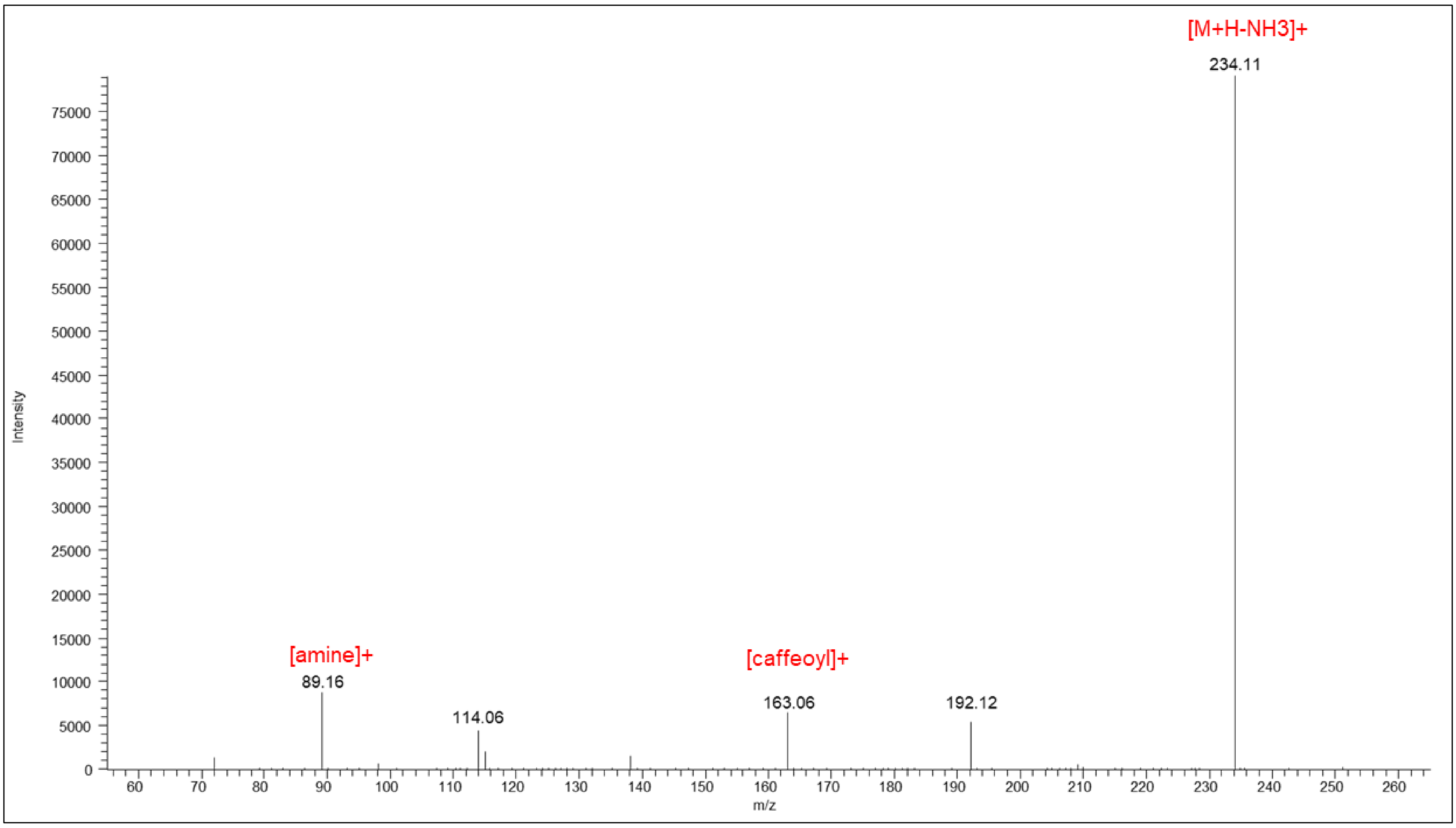

Supplementary Figure S7. Detected MS2 fragmentation pattern showing the most abundant ions produced by the 251.1388 m/z parental ion (RT 3.1), identified as n-caffeoylputrescine (M+H)+.

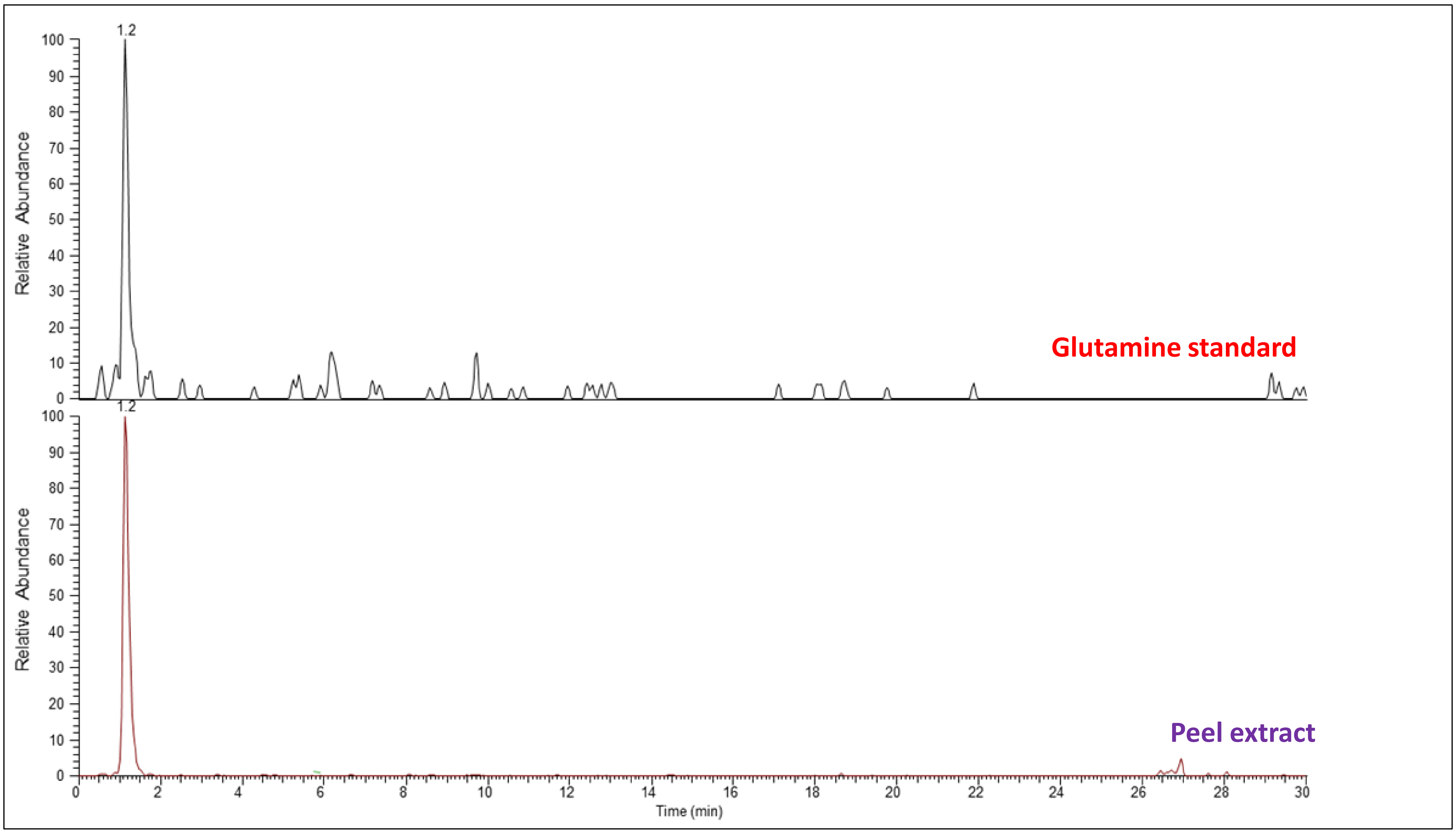

Supplementary Figure S8. Confirmation of the identity of the ion 147.0763 m/z (RT 1.2) (M+H)+, by comparison of the mobility and accurate mass of a glutamine authentic standard and peel extract, detected by the LC/ESI/MS analysis (Full MS).

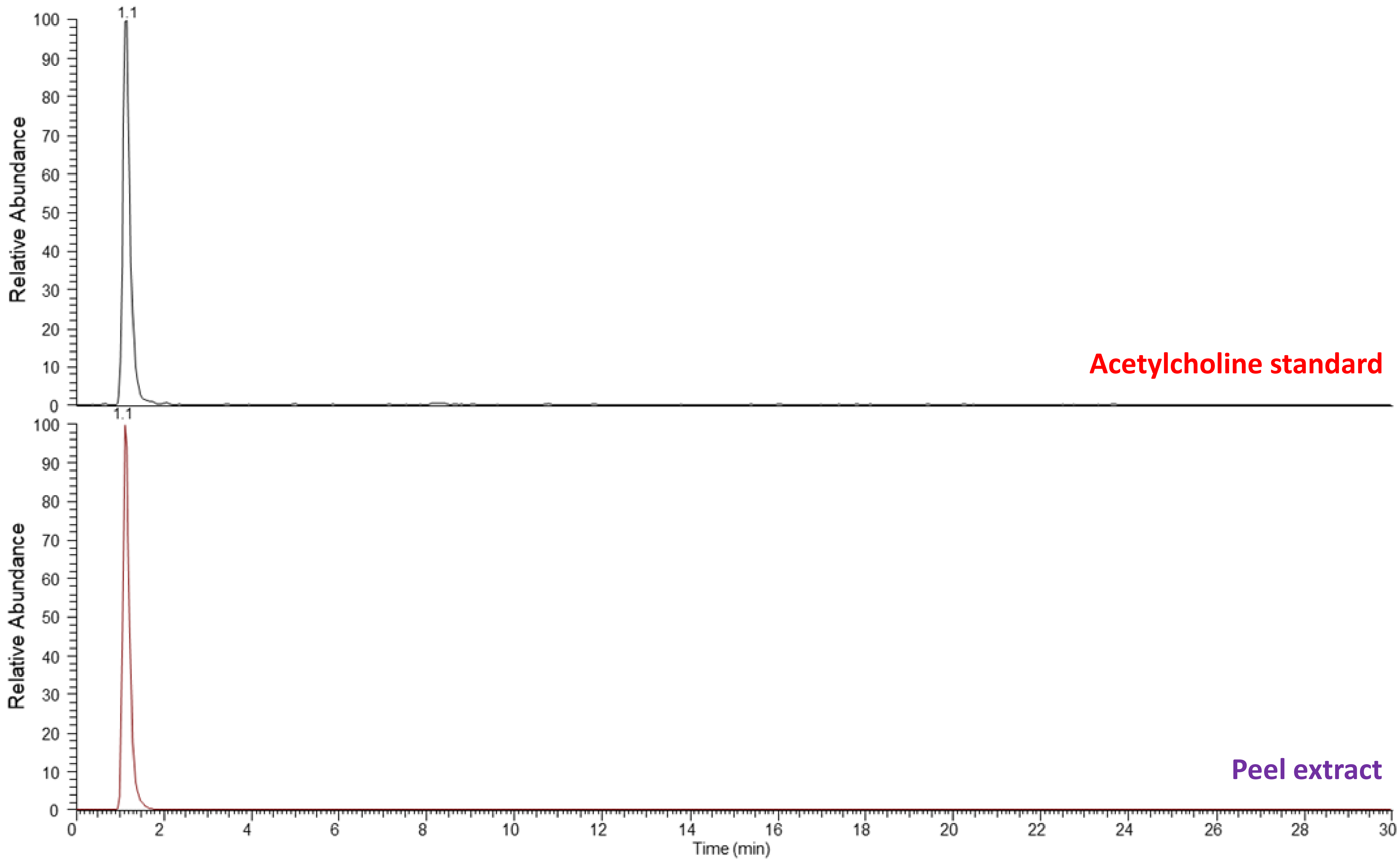

Supplementary Figure S9. Confirmation of the identity of the ion 146.1175 m/z (RT 1.1) (M+) by comparison of the mobility and accurate mass of an acetylcholine authentic standard and peel extract, detected by the LC/ESI/MS analysis (Full MS).

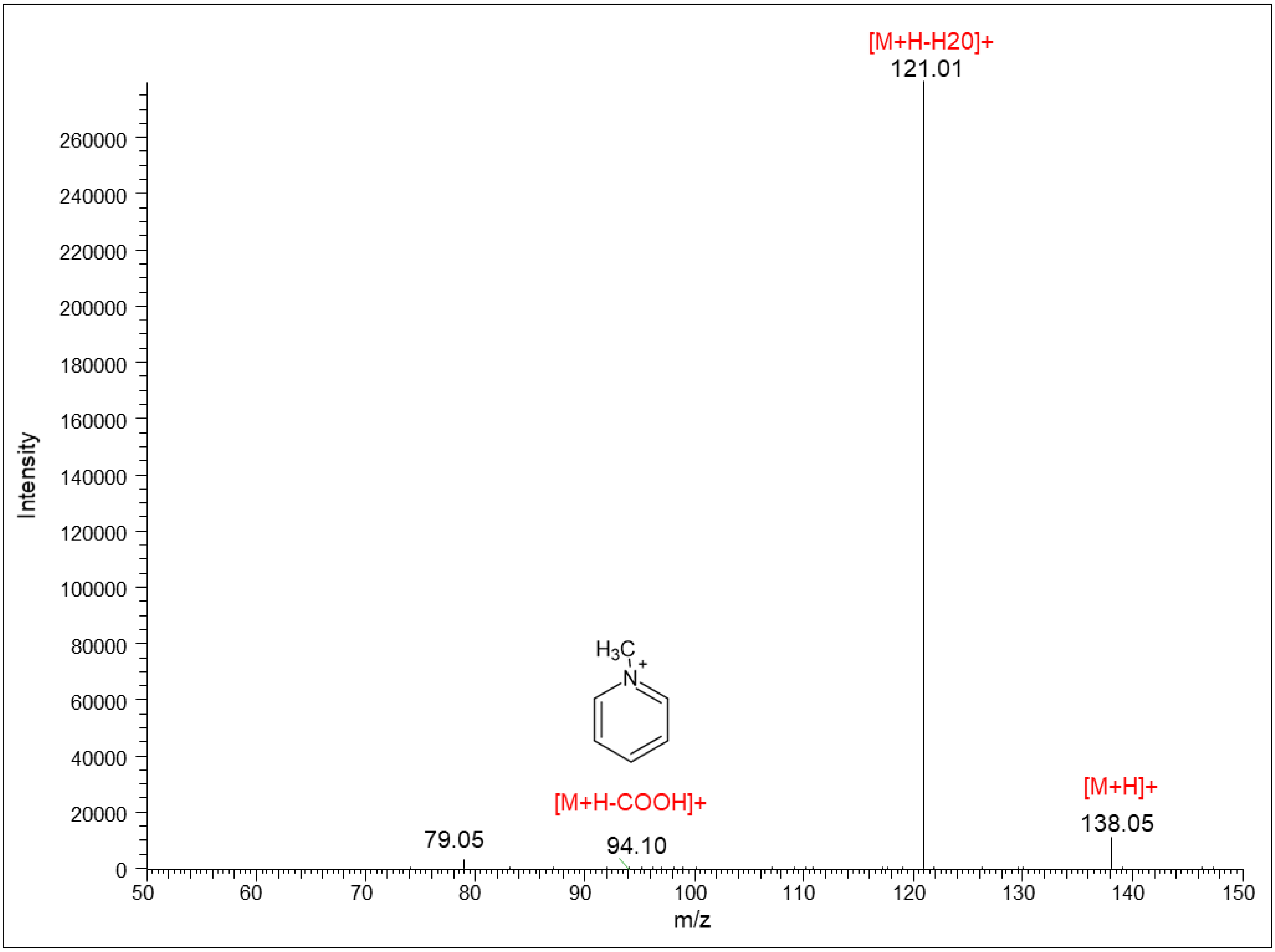

Supplementary Figure S10. MS[2] fragmentation pattern showing the most abundant ions produced by the 138.0549 m/z precursor (RT 1.1), tentatively identified as trigonelline (M+H)+.

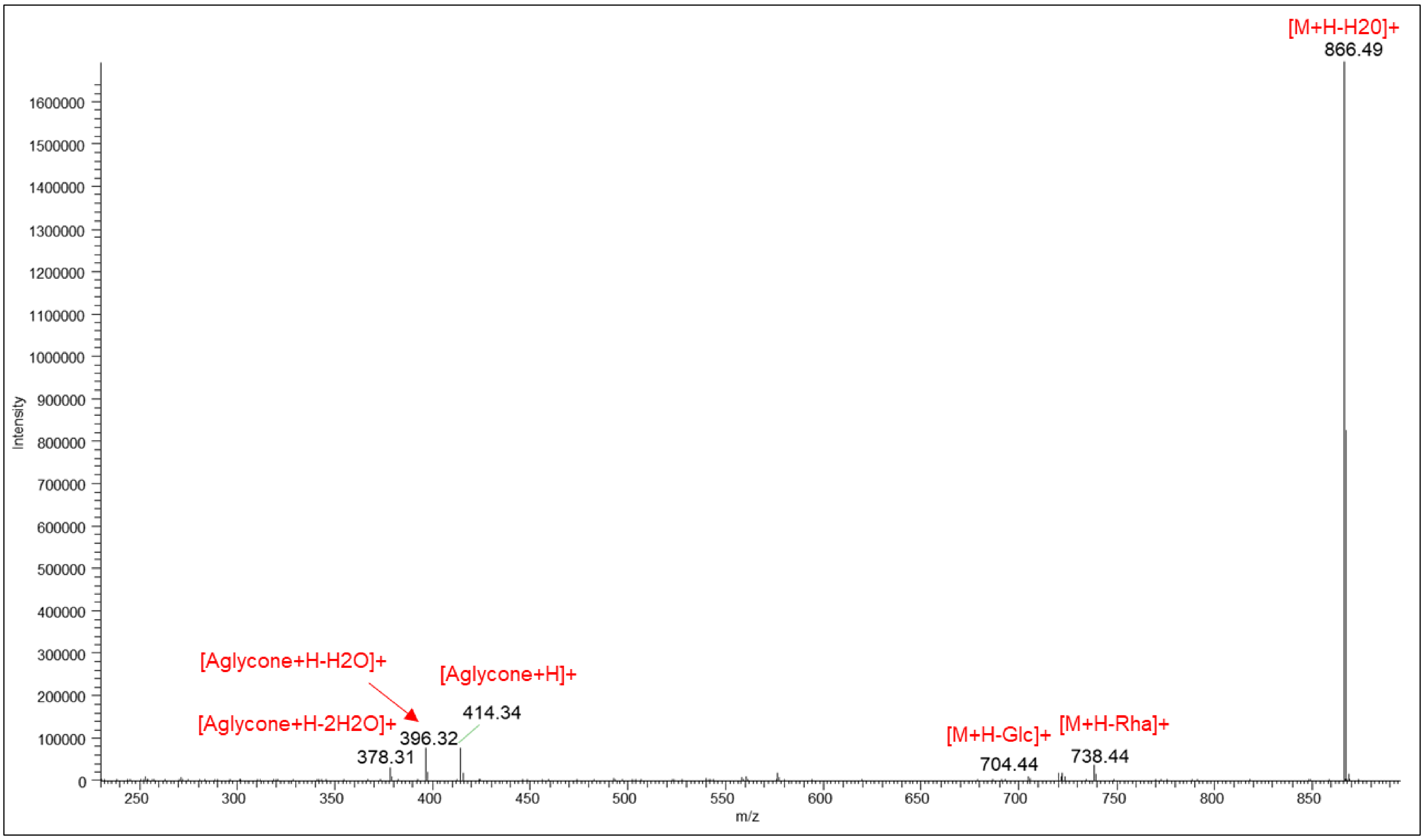

Supplementary Figure S11. MS^2 fragmentation pattern showing the most abundant ions produced by the 884.5001 m/z precursor (RT 10.2), tentatively identified as solasonine (M+H)+.

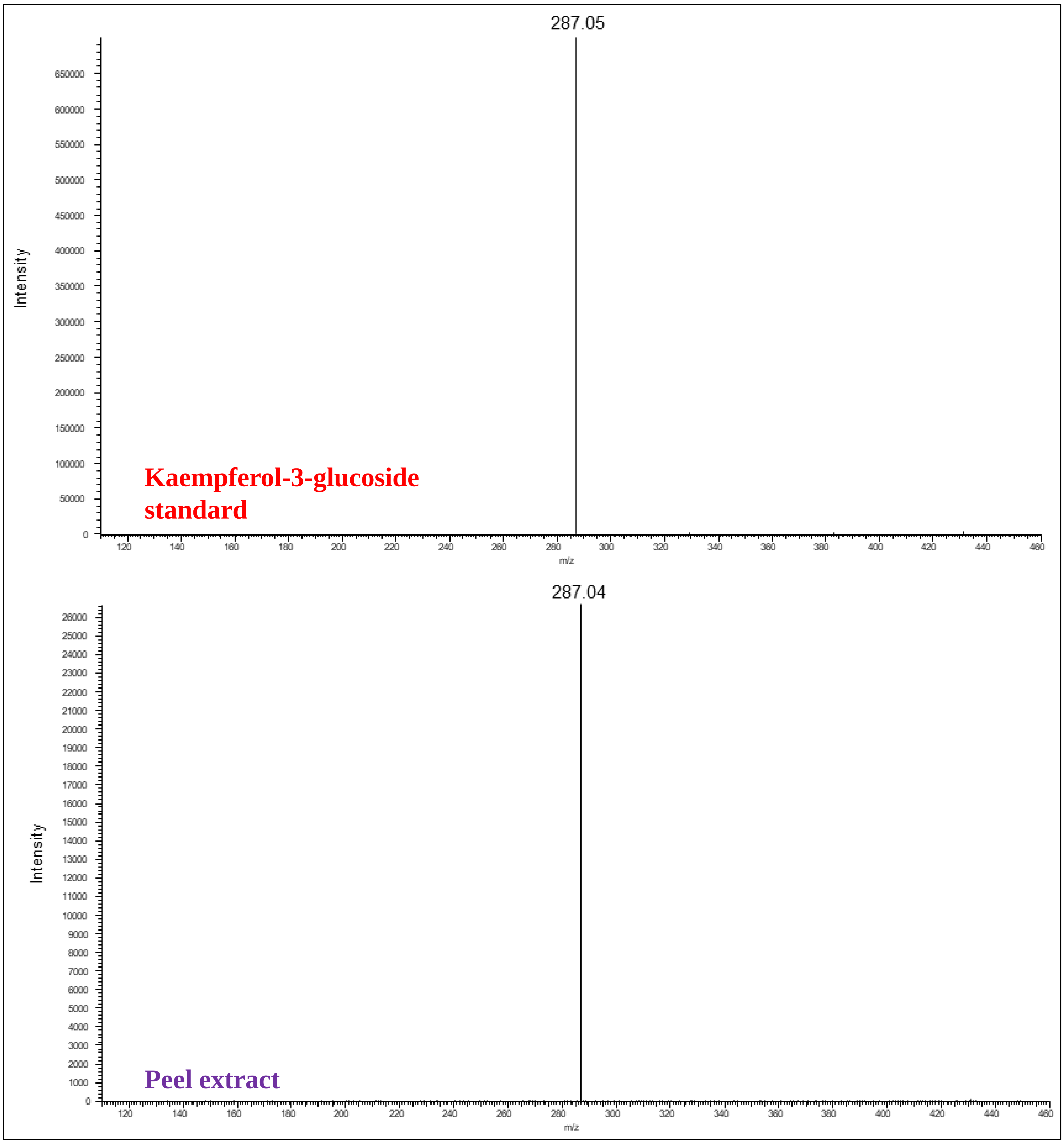

Supplementary Figure S12. Confirmation of the identity of the ion 449.1079 m/z (RT 10.1) by comparison of MS2 signals generated by parental ion in Kaempferol-3-glucoside authentic standard and peel extract.

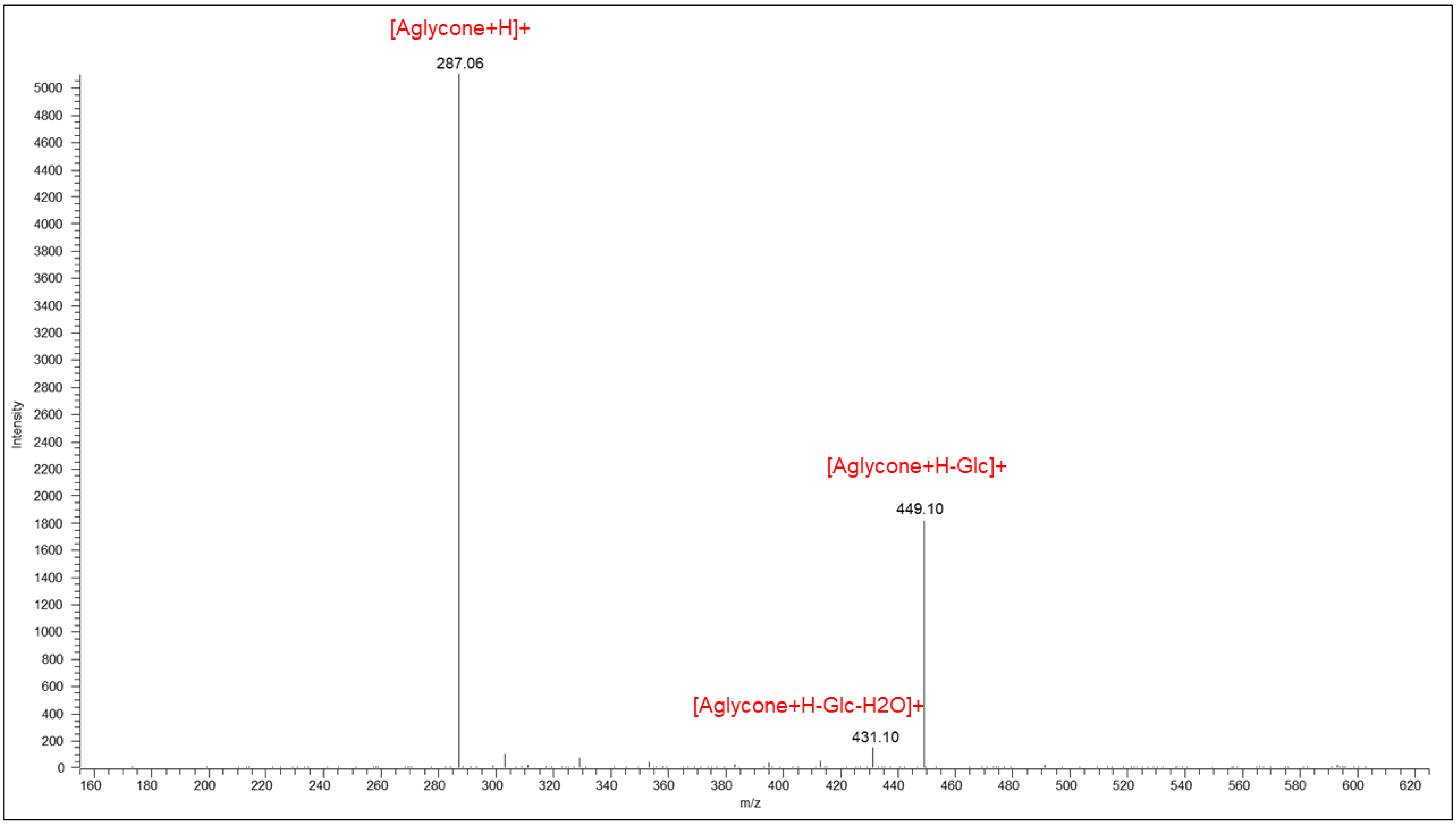

Supplementary Figure S13. MS^2 fragmentation pattern showing the most abundant ions produced by the 611.1611 m/z precursor (RT 8.6), tentatively identified as kaempferol 3-O-beta-D-sophoroside (M+H)+.

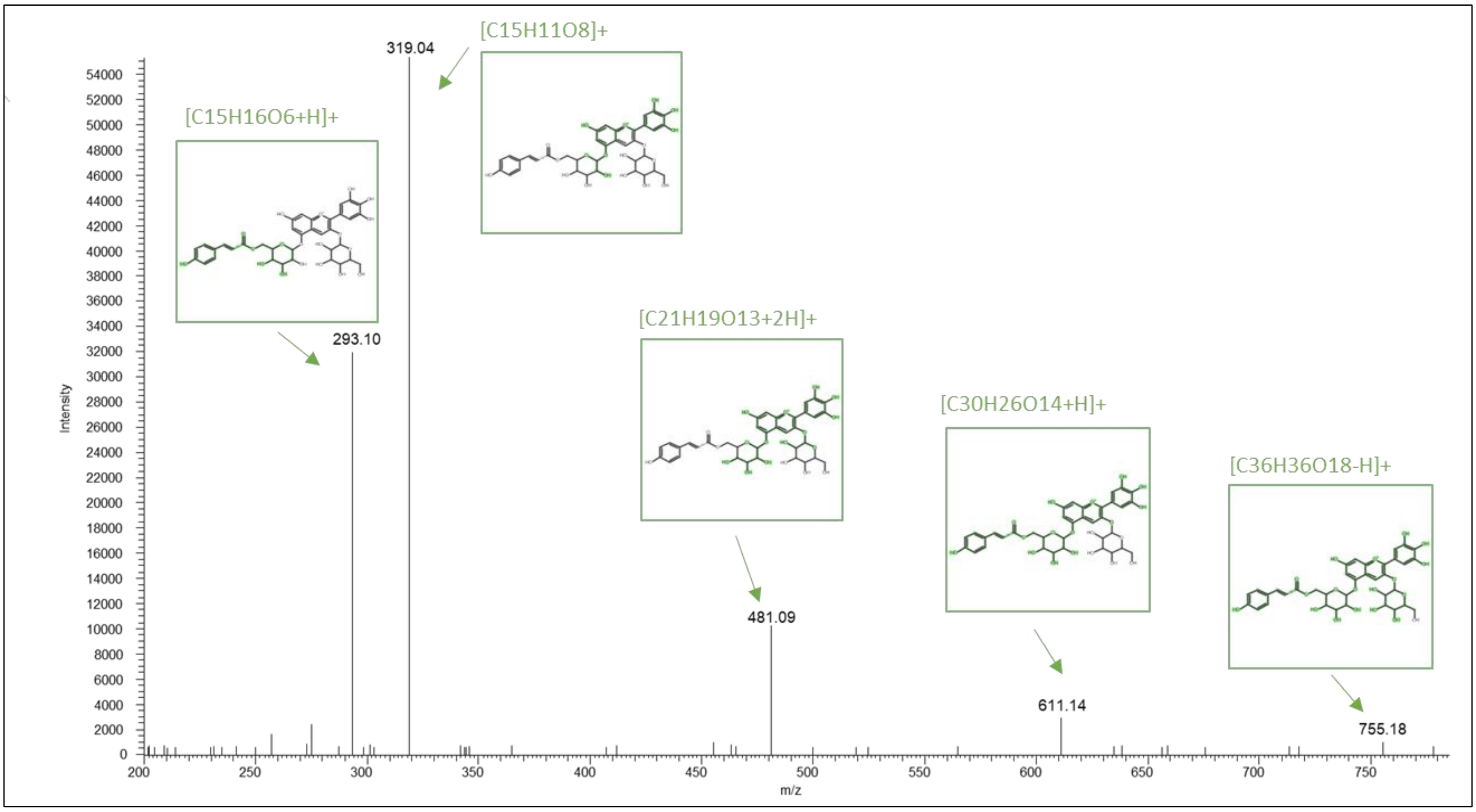

Supplementary Figure S14. MS^2 fragmentation pattern showing the most abundant signals produced by the 773.1935 m/z parent ion (RT: 11.0), identified as delphinidin 3-O-D-glucoside-5-(6-coumaroyl-D-glucoside) (M+). Green squares represent the MS^2 fragments of the parent compound predicted by MetFrag.

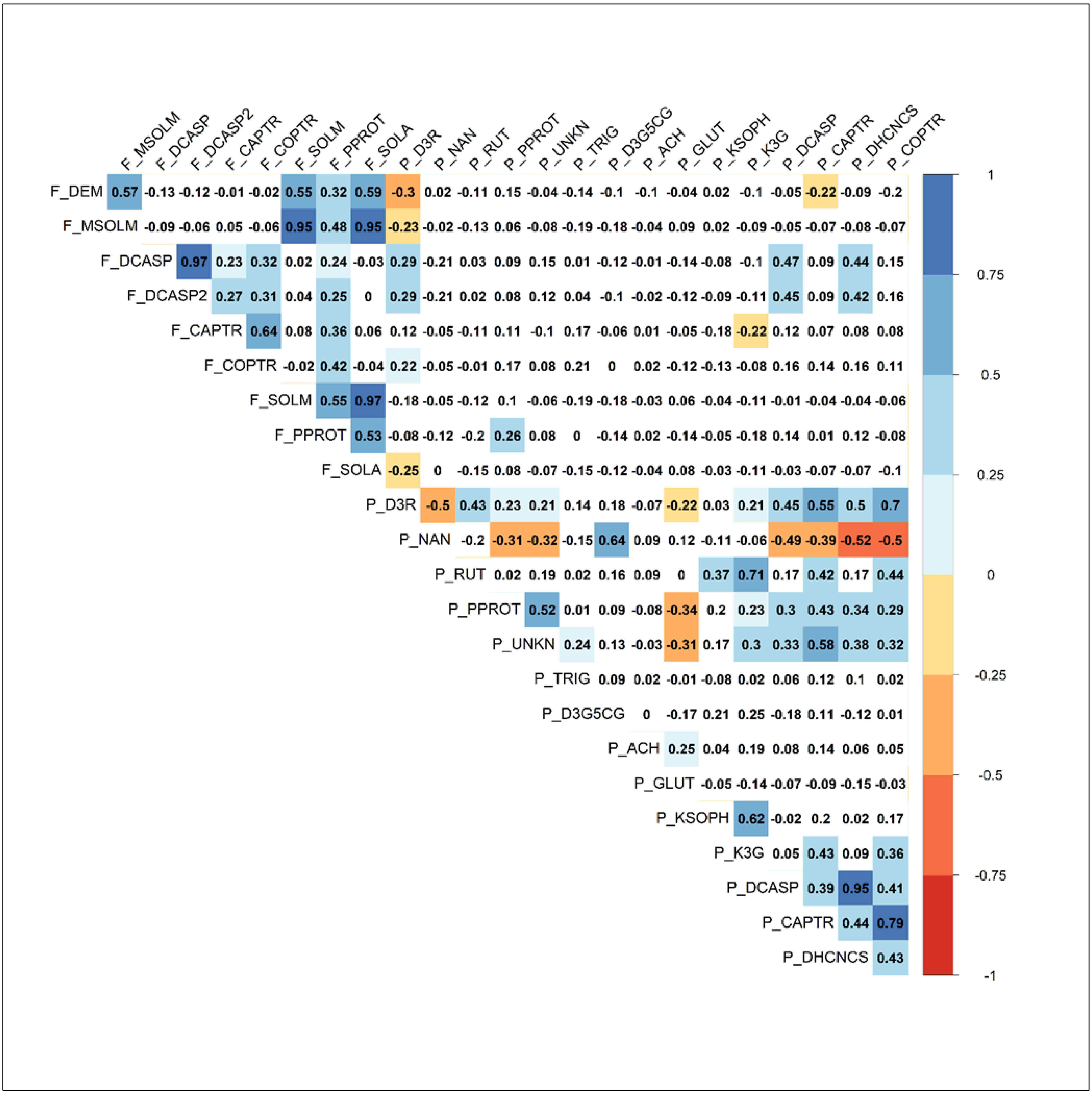

Supplementary Figure S15. Inter-trait Spearman correlations assessed in the mapping population. Colored squares show significant correlations at p<0.01.
